# Economic correlates of footbinding: Implications for the importance of Chinese daughters’ labor

**DOI:** 10.1371/journal.pone.0201337

**Published:** 2018-09-20

**Authors:** Melissa J. Brown, Damian Satterthwaite-Phillips

**Affiliations:** 1 Harvard-Yenching Institute, Cambridge, MA, United States of America; 2 Independent Researcher, Eugene, OR, United States of America; University of California San Francisco, UNITED STATES

## Abstract

**Background:**

It is a wide-spread assumption about footbinding that footbound girls and women were more of an economic burden on their families than those never bound. It is often presumed that government policies and missionary campaigns ended footbinding.

**Methods/ Objectives:**

We use regression and log-likelihood tests, with bootstrapping for confirmation, to analyze which of a series of ethnographically and historically hypothesized variables significantly correlate with footbinding. We also consider an indirect measure of government prohibitions. We analyze two large datasets based on oral surveys with elderly women of the last footbound generations from 12 inland Chinese provinces.

**Conclusions:**

Handicraft production, particularly commercial handicraft production, correlates with whether Chinese girls were subjected to footbinding before 1950. Girlhood knowledge of government prohibitions against footbinding, an indirect measure of awareness by the adults who decided whether to bind a girl’s feet, did not correlate with whether women were ever footbound. Spinning cotton thread for commercial purposes (sale, wage, direct exchange) correlated with greater daily production, with great county-level variation in quantity produced. Moreover, Chinese commercial spinners labored more years before marriage than domestic spinners.

**Implications:**

Chinese daughters—whether footbound or not—made important economic contributions to rural households, thus suggesting a need to revise our understanding of China’s gender and economic history. Further implications of our results are that research is warranted on the assumed efficacy of government prohibitions—in both rural and urban areas—and on the presumption that footbinding among elite Chinese women was unrelated to economic concerns, including handicraft production. The demonstrated economic correlates of footbinding in inland, rural China also suggest a need to reevaluate whether contemporary customs controlling and cloistering girls and women, such as female genital cutting in Africa and the threat of honor killings of girls and women in South Asia, might have economic correlates.

## Introduction

**Fig 1:**
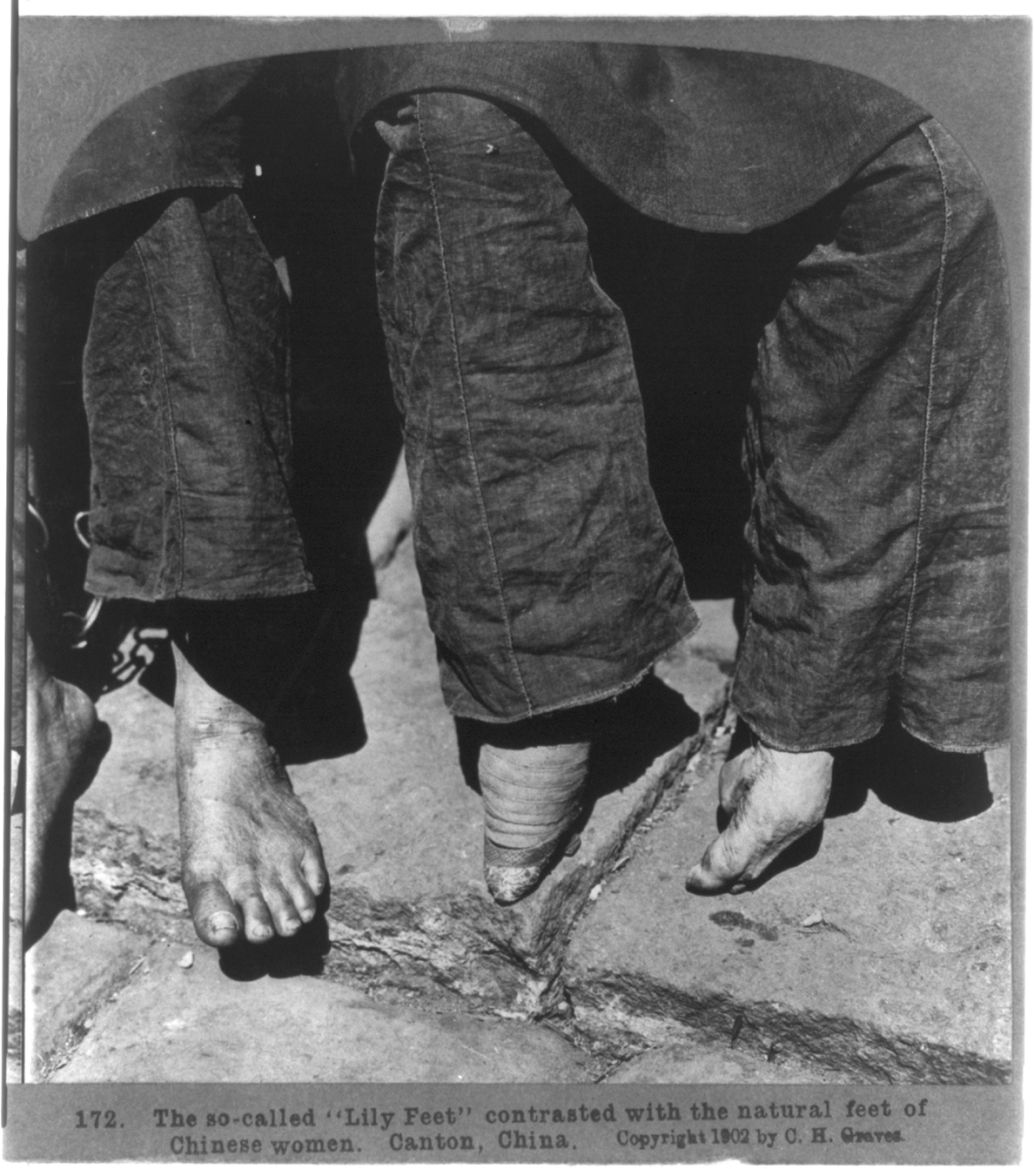
Comparison of Chinese women’s bound and natural feet, circa 1902, in Guangzhou (Canton). The woman on the left shows a bare, never-bound foot; the woman on the right shows lotus (or lily) feet, the most extreme form of bound feet, with bindings on and off. *Source*: [15].

**Fig 2:**
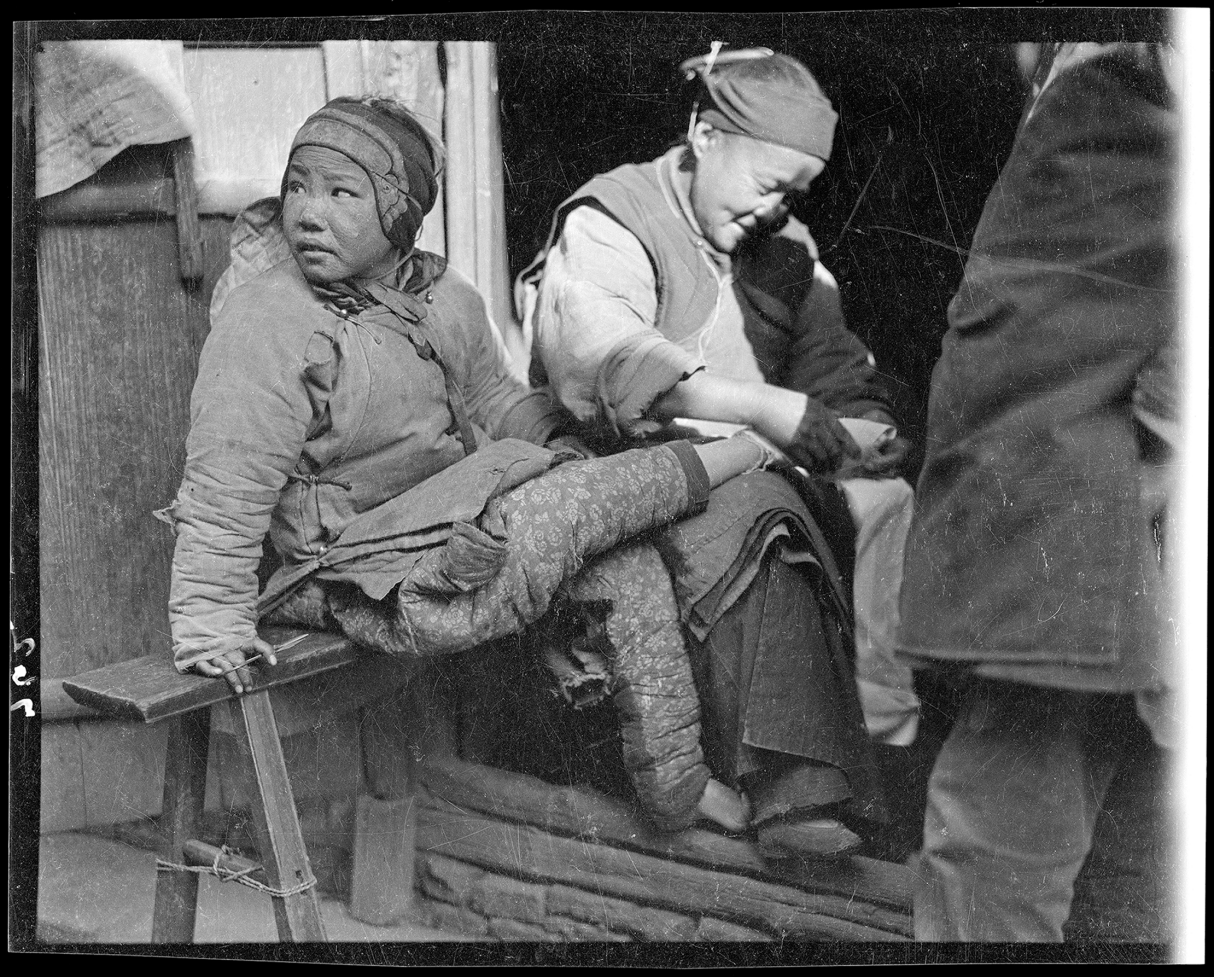
Older woman adjusting the bindings on a girl’s foot, 1917–1919, in Shilin, Zhejiang Province, China. The woman, possibly the girl’s grandmother, appears to be tightening the bindings on the girl’s right foot. Note that the woman’s shoed right foot, visible next to the girl’s shoed left foot, appears to be the same size as girl’s foot. Photo by Sidney D. Gamble, used with permission. *Source*: [16], cf. [17].

Understanding footbinding (FB), a custom which ended in China by the mid-20^th^ century, is important today for two primary reasons (see Figs 1 and 2). First, it is widely used to illustrate that cultural beliefs can override economic interests, from scholarly works to high school text books (e.g., [1–3]). More specifically, FB is said to show that cultural beliefs can disempower and cloister girls and women, despite economic hardships that result to families from the presumed loss or restriction of female labor contributions (e.g., [4–8]). Second, models based on the assumption that social-mobilization efforts ultimately halted the custom of FB are being promoted as a means to end contemporary practices of female genital cutting [9] and honor killing of girls and women [2]. Thus, the evidence we present that FB had strong economic correlates suggests the need to reevaluate the complex relations among economic development, empowerment of girls and women, and cultural beliefs (cf. [10–14]).

There are three long-standing assumptions of FB, all of which evidence now suggests are mistaken. First, reformers, scholars, and footbound women assumed FB was a custom derived from cultural beliefs about beauty (or sexuality) and related to girls’ marriage prospects in China’s patriarchal, patrilineal society where—in late imperial times as well as today—there was a demographic shortage of women on the marriage market [18]. Second, FB was long assumed to severely limit the ability of Chinese girls and women to contribute economically to their households [5–8, 19–23]. Third, the social-engineering efforts of reform-minded activists and political leaders, some Chinese and some Western, were assumed to have ended FB (see Appendix A: Historical Context; Figs A–J in S1 Images: Spinning, Weaving & Cloth; Figs A–C in S2 Images: Variation in Bound Feet). Previous research shows that the first assumption is false [18]. For most women, throughout most of the early 20^th^ century, FB made no significant difference in their ability either to marry at all or their ability to marry “up” to an economically better-off household. Moreover, FB ended despite women’s enduring but mistaken belief that FB would lead to a better marriage.

In this paper, we examine whether evidence supports the second assumption and consider indirect evidence regarding the third assumption. We test the following hypotheses: (a) premarital hand-labor correlates with FB and an absence of prohibitions does not; (b) the correlation between hand-labor and FB existed for multiple generations; and (c) handicraft production in rural homes for commercial purposes (sale, wage, or direct exchange of goods) correlates with the amount of labor a girl contributed to her premarital household. We refer to the models associated with these hypotheses as (a) “FB predictor,” (b) “generational,” and (c) “labor” models.

## Data

**Fig 3:**
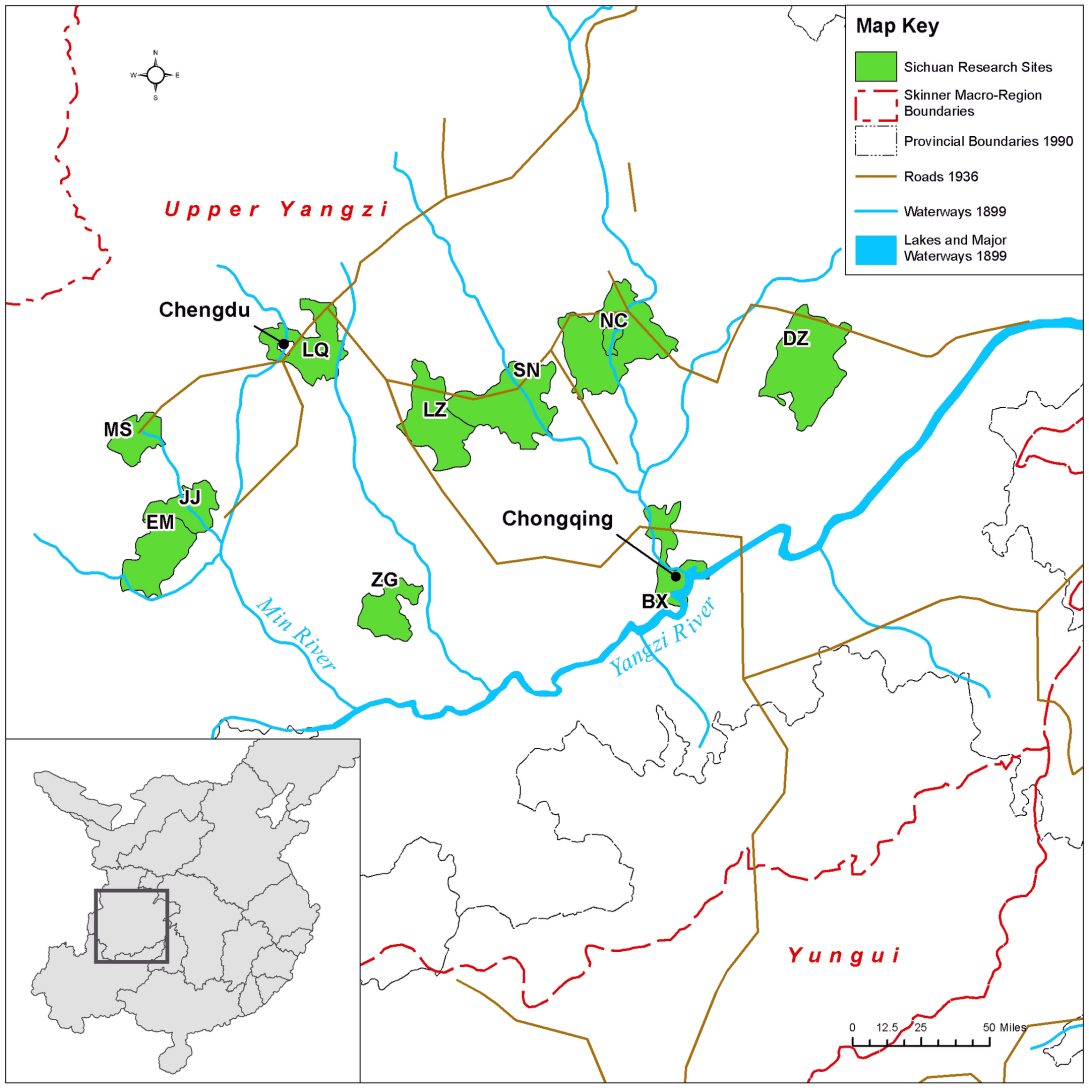
Map of Sichuan research sites in relation to macroregions and to shipping and trucking transport routes circa 1936. Map created by the Center for Geographic Analysis, Harvard University (based on [24–29]).

**Fig 4:**
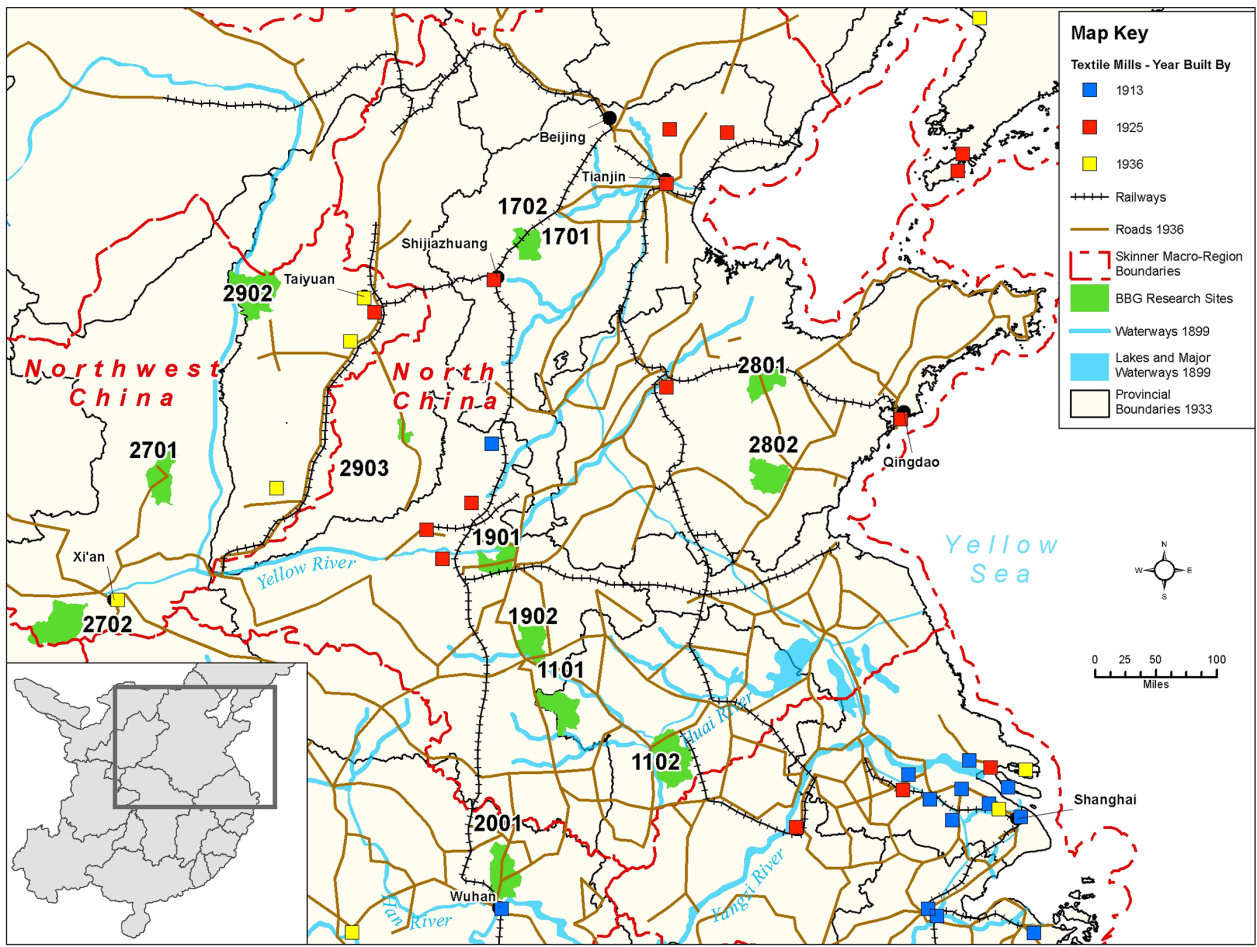
Map of Northern research sites in relation to macroregions; to shipping, trucking, and rail transport routes; and to textile mills circa 1936. Map created by the Center for Geographic Analysis, Harvard University (based on [24–29]).

**Fig 5:**
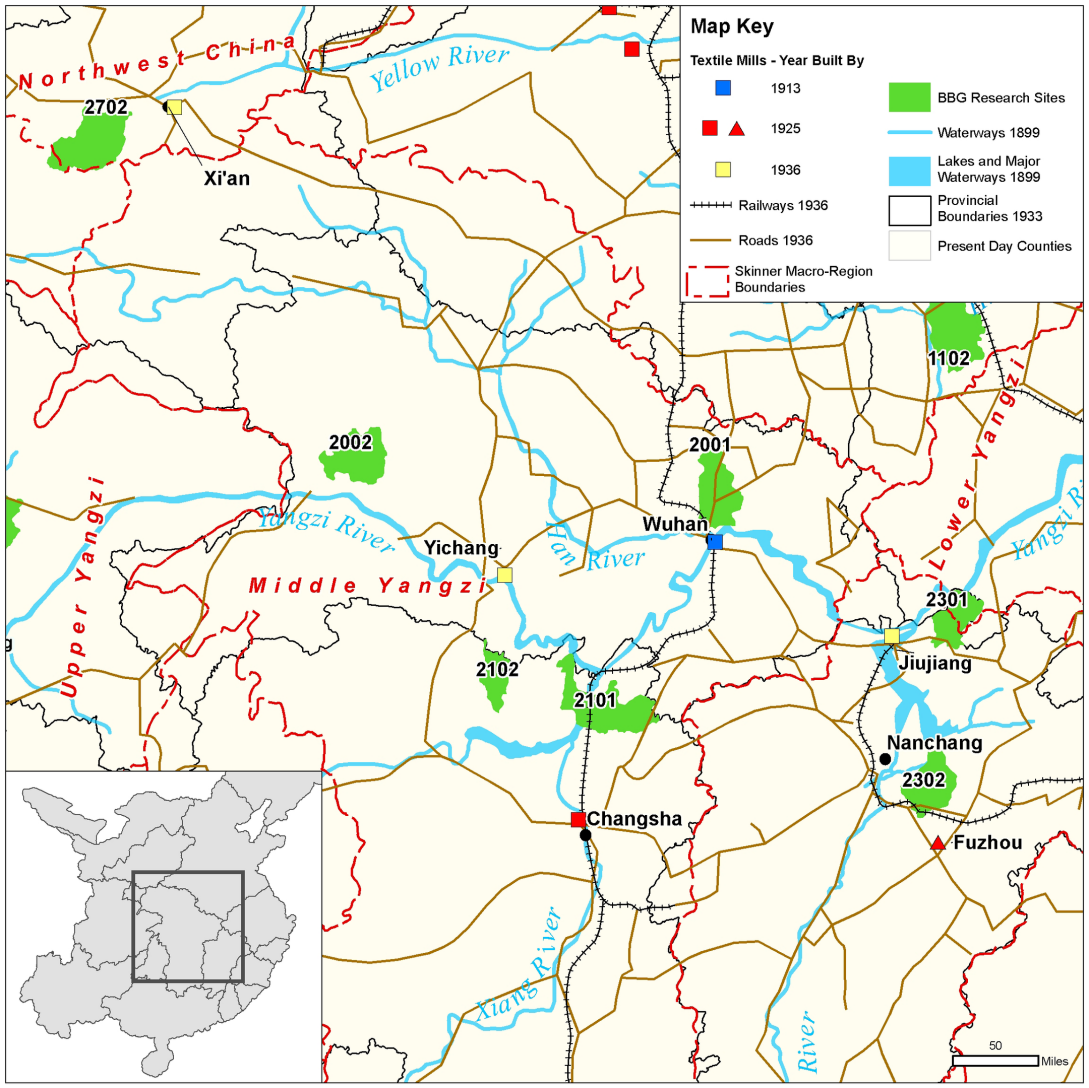
Map of Central research sites in relation to macroregions; to shipping, trucking, and rail transport routes; and to textile mills circa 1936. Map created by the Center for Geographic Analysis, Harvard University (based on [24–29]).

**Fig 6:**
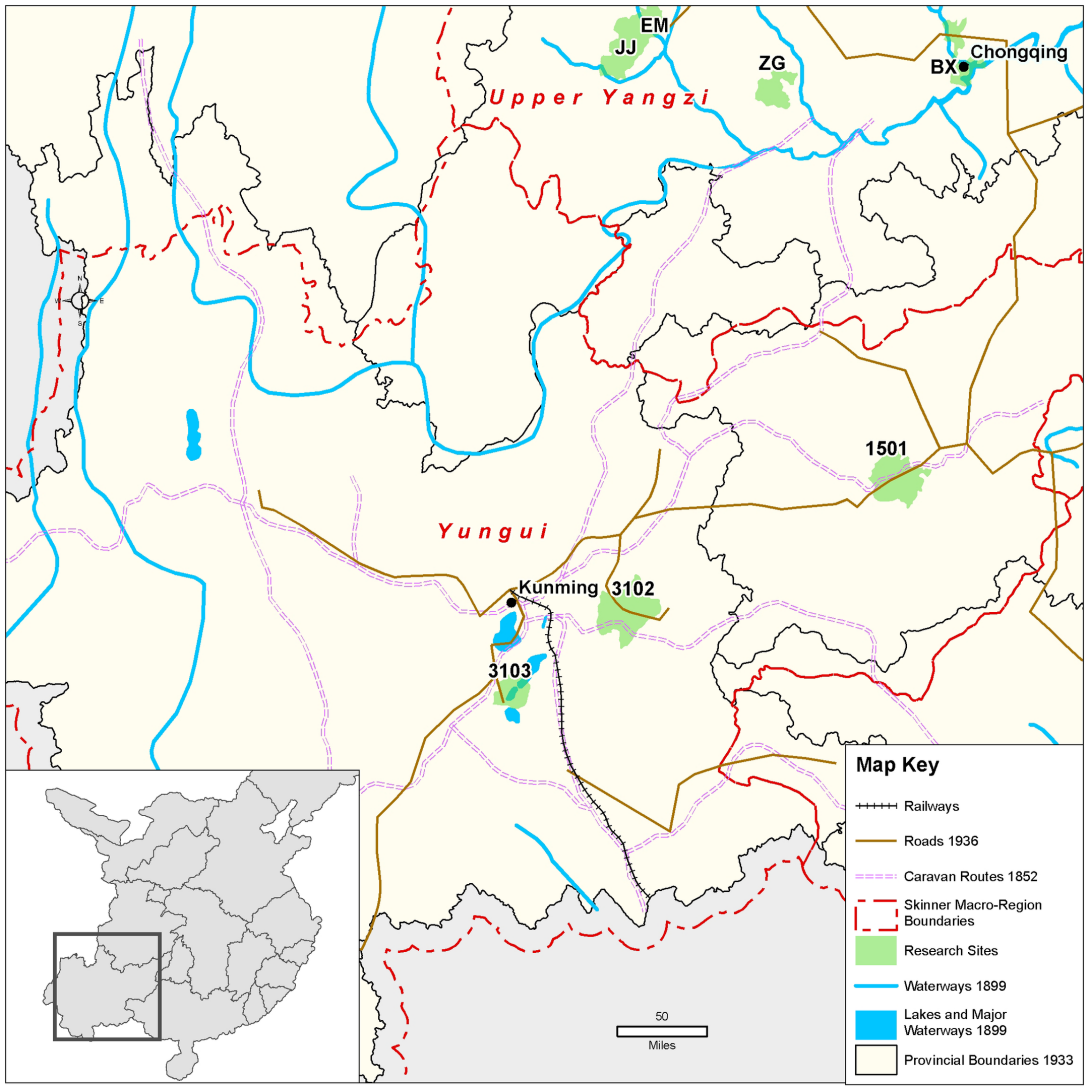
Map of Southwestern research sites in relation to macroregions and to shipping, trucking, rail, and caravan transport routes circa 1936. Map created by the Center for Geographic Analysis, Harvard University (based on [24–29]).

The quantitative information about Chinese FB presented here comes from collaborative research using orally administered structured interviews with 4973 women in 10 counties in Sichuan Province (gathered during the early 1990s, the “Sichuan dataset” [18, 22]; Fig 3) and 2710 women in 20 counties in 11 other northern, central, and southwestern inland Chinese provinces (gathered 2006–2011, the “BBG dataset” [18, 23]; Figs 4–6; S1 Dataset). These datasets focus on inland, primarily rural areas because it was in these locales that significant numbers of living women could still be found at the end of the 20^th^ century who had experienced footbinding (footbinding ended decades earlier in coastal areas and in urban centers; Appendix A).

Both datasets provide information for each woman about: the wealth and education of her natal household; the labor she performed while living there, focusing on handicraft production and field agriculture; and her experience of footbinding. There are very few women of high social class in these datasets. Women who reported that both they and their father were literate or educated make up less than 4 percent of the combined datasets (cf. Table 1). Such multigenerational education represents the highest socioeconomic class—an elite, including high officials and wealthy industrialists, who were a small percentage of the early twentieth-century Chinese population. Nevertheless, these datasets may underrepresent elite women because the data were collected among women still living in inland, rural areas during the 1990s (for the Sichuan dataset) and early 2000s (for the BBG dataset). The elite from these areas left, if they could, during the wars (1937–1949) or the 1980s. The BBG dataset differs from the Sichuan dataset by including additional information on FB prohibitions, each woman’s mother, daily quantities spun in the natal household, and years of labor contributed to the natal household. (Data collection, ethics statement, datasets, variables, and regions are more fully explained in Appendix B: Detailed Materials and Methods.)

**Table 1:** Descriptive statistics of categorical variables.

variable	yes	no	*n* (# who answered)	NA (no answer)
ever footbound	4287 (57.3%)	3194 (42.7%)	7481	40
literate	“half”: 707 (9.7%) fully: 251 (3.5%)	6300 (86.8%)	7258	263
any education	651 (24%)	2058 (76%)	2709	4812
mother any education	275 (3.7%)	7122 (96.3%)	7397	124
father any education	2134 (29.7%)	5061 (70.3%)	7195	326
domestic hand labor	3541 (49.1%)	3667 (50.9%)	7208	313
commercial hand labor	3062 (43.1%)	4043 (56.9%)	7105	416
any agricultural (field) labor	4053 (55.5%)	3246 (44.5%)	7299	222
mother footbound	2097 (90%)	231 (9.9%)	2328	5193
mother spin anything	443 (46%)	520 (54%)	963	6558
heard of prohibition	1496 (67%)	736 (33%)	2232	5289
own a loom	794 (32%)	1688 (68%)	2482	5039
feet bound by mother	3667 (86.6%)	567 (13.4%)	4234	3287

BBG and Sichuan datasets combined; women born before 1943. (Percentages calculated in terms of the number of women who answered the question.)

**Fig 7:**
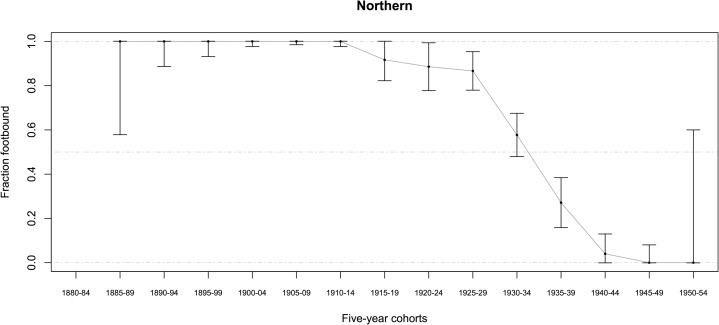
Percentage of Chinese women ever footbound in 11 Northern rural counties, by birth cohort (*n* = 568; error bars indicate the 95% CI). Source: BBG data, women reporting on themselves and their mothers.

**Fig 8:**
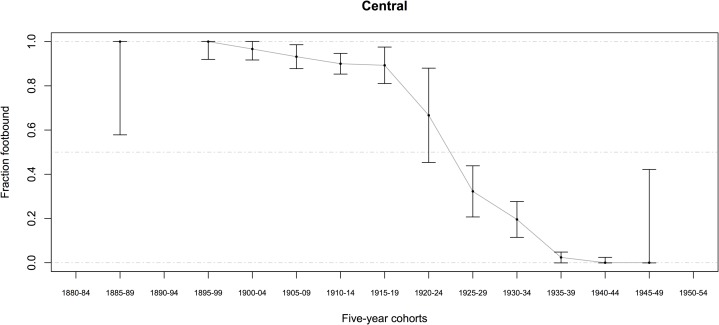
Percentage of Chinese women ever footbound in 6 Central counties, by birth cohort (*n* = 780; error bars indicate the 95% CI). Source: BBG data, women reporting on themselves and their mothers.

**Fig 9:**
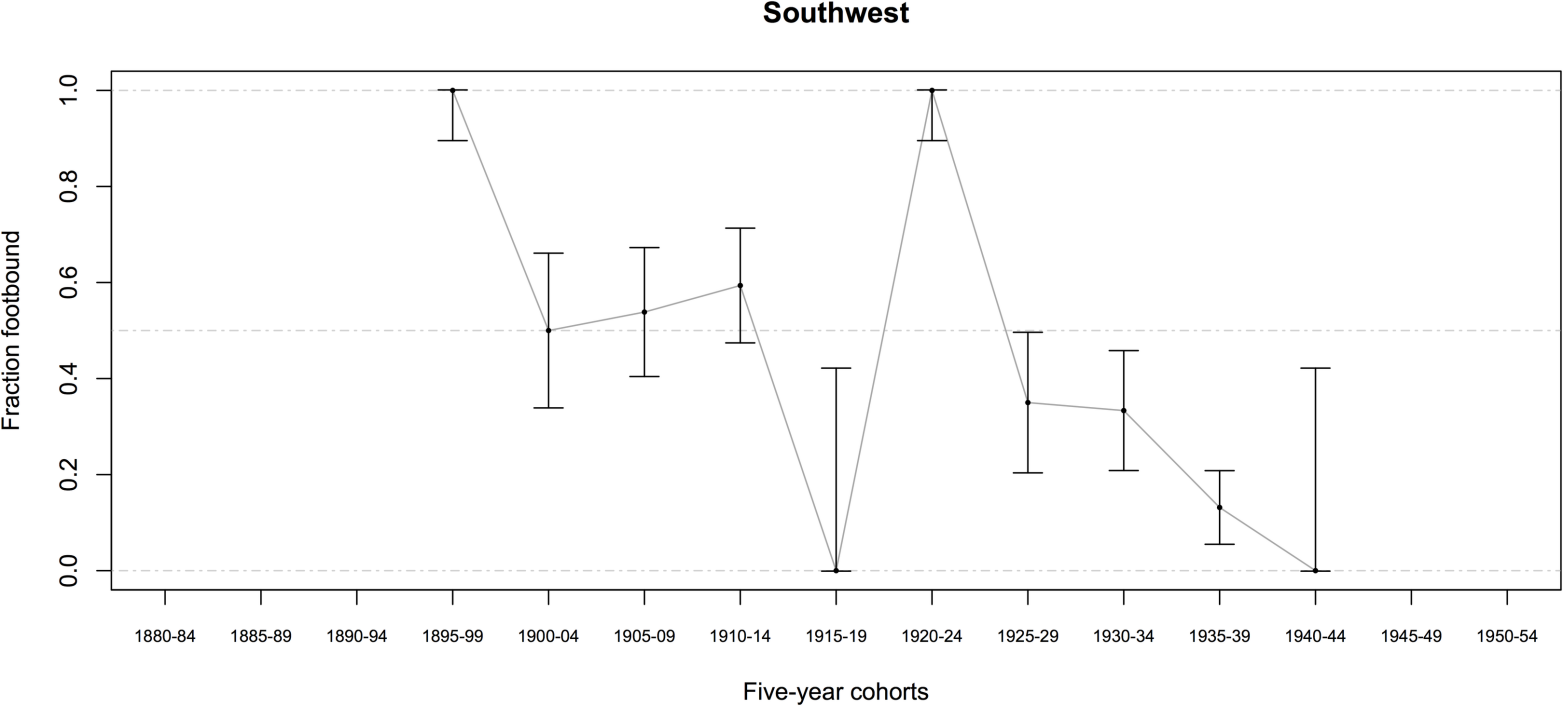
Percentage of Chinese women ever footbound in 3 Southwestern rural counties, by birth cohort (*n* = 350; error bars indicate the 95% CI). The spike in the 1920–1924 cohort is probably due to sampling error (only 12 women contributed data to this point, all of whom were footbound). Source: BBG data, women reporting on themselves and their mothers.

**Fig 10:**
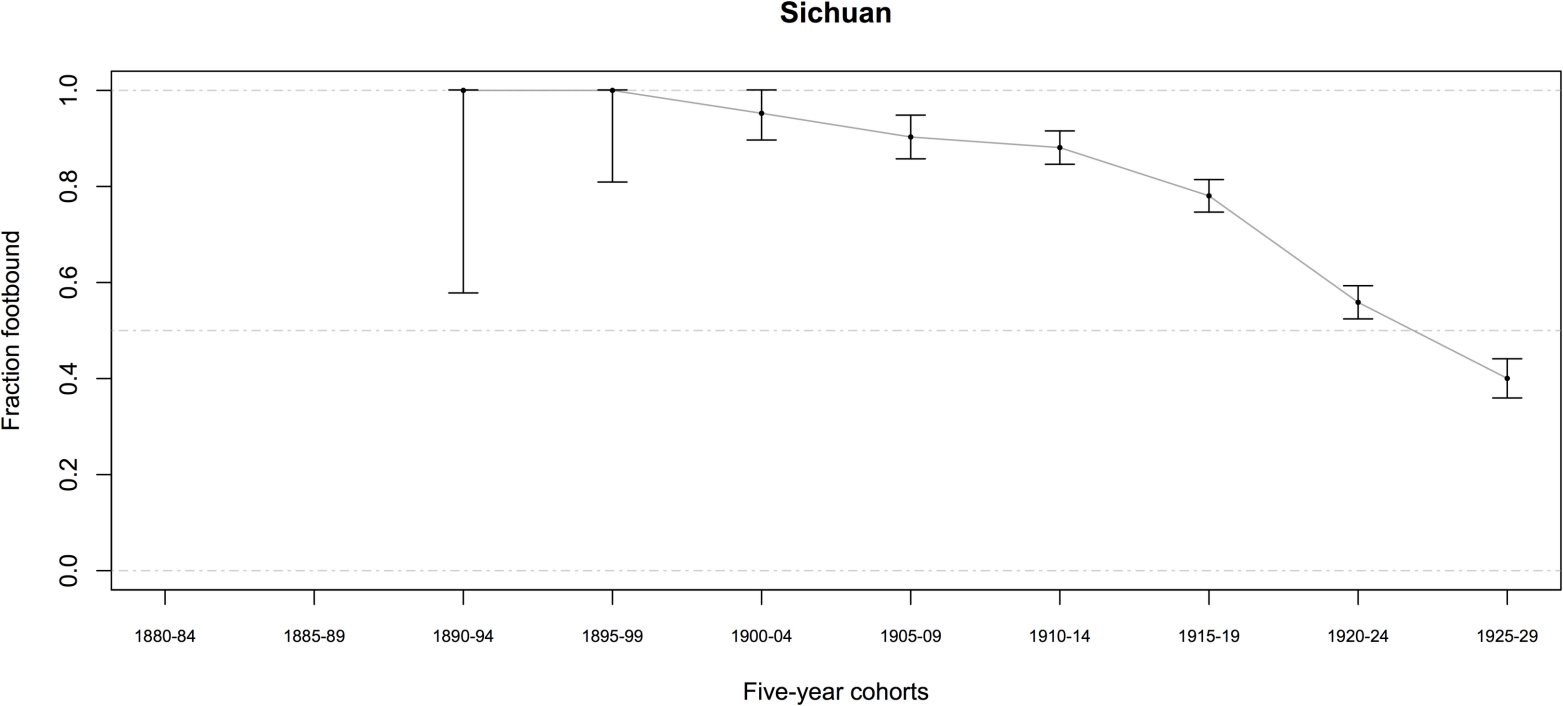
Percentage of Chinese women ever footbound in 10 Sichuan counties, by birth cohort (*n* = 2489; error bars indicate the 95% CI). Source: Sichuan data, women reporting on themselves.

Most women (in both datasets combined) were born between 1905 and 1942 and lived in rural communities most of their lives (some in Sichuan were born as early as the 1890s). Among women born before 1943, 57.3 ± 1.1 percent (95% CI, *n* = 7481) had ever had their feet bound, even for a brief time (Tables 1 and 2) (*n* indicates the number of women who answered the relevant question, and CI were determined by treating percentages as the outcome of a binomial random variable, with $\hat{p}=\frac{successes}{failures}$). The percentage of women ever footbound (fb), although varying by county, generally declined from 1900 to 1950 (Figs 7–10). Women born before 1943 reported their feet were bound at a mean age of 6.4 *sui* or approximately 5 years old, and about 87 percent of fb women reported their feet were bound by their mothers (Tables 1 and 2).

**Table 2:** Descriptive statistics of major variables used.

variable	*n* (# who answered)	mean	median	SD
ever footbound*^a^	7481	0.57	1	0.49
birth year	7521	1923.54	1924	8.77
rural wealth index^b^	7328	2.24	3	1.57
years footbound^c^	3251	5.40	4	5.92
marriage year	7496	1941.68	1942	9.79
age at footbinding^d^	4231	6.39	6	2.92
age at learning to spin ^d^	1414	12.42	12	4.35
age at marriage ^d^	7496	18.15	18	3.18
amount of daily cotton spun^e^	463	0.28 (169g)	0.22 (133g)	0.22
years of premarital labor	1405	6.27	6.5	4.98

BBG and Sichuan datasets combined; women born before 1943.

* Ever footbound, a categorical variable, is included here to show standard deviation.

^a^ fb = 1, never bound = 0

^b^ integer-values 0–4; family owned land = +2, owned house = +1, owned draft animal = +1

^c^ the number of years of age (in *sui*) from first binding to (first) unbinding

^d^ age in *sui*

^e^ amount in catties (grams); 1 catty = 603.277 grams

Only the BBG dataset provides information on unbinding—when an individual undid the binding cloth to “let out” her feet, an action possible if the arch was unbroken (see Fig A in S2 Images). Unfortunately, the unbinding variable conflates temporary unbinding, when feet are let out for a few weeks, months, or even years before rebinding, with permanent unbinding, when the feet were never rebound. Qualitative reports show that many women unbound their feet to avoid a fine, part of the implementation of some prohibitions (just as in the documentary *Small Happiness*, which was filmed about 1980 [30]), or to flee the invading Japanese army, but many rebound after the danger passed. These comments reveal that, for many rural, inland Chinese women during the first half of the twentieth century, FB was not a single event but a cycle of binding, unbinding, and rebinding.

There was a difference between the BBG and Sichuan datasets in the proportion of women involved in domestic and commercial handicraft production before marriage. Handicrafts include spinning, weaving, and embroidering textiles; making shoes, clothes, nets, baskets, and mats; and raising silkworms. We defined hand labor as commercial if it produced goods for sale, wage, or direct exchange, and as domestic if it produced goods for use within the household. Most women in our Northern, Central, and Southwest regions (the BBG dataset) reported domestic handicraft production in their premarital homes (*n* = 2099), though in the Southwest, the majority of women did both domestic and commercial hand labor (Tables 3–5). By contrast, in Sichuan (*n* = 4969; Table 6), 43.6 percent of women did commercial hand labor, but more women reported that they were not involved in handicraft production of any sort (37 percent) than reported doing domestic hand labor (32.7 percent). We focus on *premarital* handicraft production and labor in order to examine the relation between labor and footbinding in households that were making the decision on whether to bind girls’ feet (the average age of footbinding was about 5 years old, Table 2). Moreover, in the BBG dataset, we are also able to assess footbinding and at least one form of hand labor one generation prior by considering premarital households, since far more women were able to report whether their mothers did any spinning than whether their mothers-in-law did (Table 7).

**Table 3:** Northern women conducting commercial and domestic handicraft production.

		commercial		
		yes	no	total
**domestic**	**yes**	358 (41.7%)	403 (46.9%)	761 (88.6%)
	**no**	31 (3.6%)	67 (7.8%)	98 (11.4)%
	**total**	389 (45.3%)	470 (54.7%)	859 (100%)

BBG database, Northern sites only; women born before 1943

**Table 4:** Central women conducting commercial and domestic handicraft production.

		commercial		
		yes	no	total
**domestic**	**yes**	233 (26.1%)	491 (55.1%)	724 (81.2%)
	**no**	17 (1.9%)	151 (16.9%)	168 (18.8%)
	**total**	250 (28.0%)	642 (72.0%)	892 (100%)

BBG database, Central sites only; women born before 1943

**Table 5:** Southwest women conducting commercial and domestic handicraft production.

		commercial		
		yes	no	total
**domestic**	**yes**	200 (57.5%)	91 (26.1%)	291 (83.6%)
	**no**	23 (6.6%)	34 (9.8%)	57 (16.4%)
	**total**	223 (64.1%)	125 (35.9%)	384 (100%)

BBG database, Southwest sites only; women born before 1943

**Table 6:** Sichuan women conducting commercial and domestic handicraft production.

		commercial		
		yes	no	total
**domestic**	**yes**	663 (13.3%)	962 (19.4%)	1625 (32.7%)
	**no**	1506 (30.3%)	1838 (37.0%)	3344 (67.3%)
	**total**	2169 (43.7%)	2800 (56.3%)	4969 (100%)

Sichuan database only; women born before 1943

**Table 7:** Footbinding and spinning among mothers.

Mothers		did any spinning		
		yes	no	total
**footbound**	**yes**	414 (43.3%)	460 (48.1%)	874 (91.4%)
	**no**	28 (2.9%)	54 (5.7%)	82 (8.6%)
	**total**	442 (46.2%)	514 (53.8%)	956 (100%)

BBG dataset; mothers of *all* women interviewed (including b. > 1942)

## Modeling

To test our hypotheses, we performed regressions and log-likelihood tests. In order to compare models with different underlying datasets, we used the adjusted *r*^2^ as our goodness-of-fit criterion. We used bootstrapping as additional verification to make the models more robust and to minimize the influence of sampling bias on significant variables due to clustered errors. Model reduction was done by iterative deletion and addition of predictors, and the best-fitting models are presented here. All statistical analyses were conducted with R (version 3.0.2; [31]). For logistic regression models, we computed *r*^2^ according to the method in Nagelkerke [32], which we carried out in R via the nagelkerkeR2 function in the fmsb package [33] and computed the adjusted *r*^*2*^ from that value.

For all the FB-predictor models (A1–A6) and all the labor models (C1–C6), we ran separate versions, one including all ever-fb women, and one excluding those who were bound for less than a year of age (this information was not available for the generational model, B1). Both versions yielded largely the same significant predictors (at *α* = 0.05; Table A in S1 Analysis; for differences at the regional level [models A3–A6] and county level [C2–C5], see the discussion in the S1 Analysis and S2 Analysis, respectively). Here, we present the models for those fb one year of age or more because these models yielded the better fit. (The models using all ever-fb women are in the S1 Analysis: Regional Results for the FB-Predictor Model [A3–A6]. The variables for all models are explained in Appendix B.) The “fb-one-year” models better assess correlates of long-term FB status by excluding women who bound less than one year of age (n = 404).

### A. FB predictor models (A1–A9)

We performed a logistic regression with FB status as the response variable (1 = bound at least one year, 0 = never bound). Commercial premarital hand-labor experience (yes/no) and domestic premarital hand-labor experience (yes/no) were the hypothesized correlates. We also included the following covariates in the complete models for the BBG dataset (A1) and the Sichuan dataset (A2): county, birth year, education (some/none), literacy (illiterate, half-literate, literate), mother’s and father’s education (some/none), premarital agricultural labor experience (yes/no), a natal-family wealth index (integer-values 0–4) ([18]; Appendix B). For the FB-predictor models using the BBG dataset (A1, A3–A5), we also included mother’s fb status (yes/no), and knowledge in girlhood of a prohibition against FB (yes/no). FB-predictor models A3–A6 examine variation at the regional level, using the same variables as above except they distinguished three types of hand labor (spinning, weaving, and “other”). (Models A3–A6 are presented in S1 Analysis.) Interaction terms were not considered due to insufficient degrees of freedom. In the final models for the BBG dataset (A1) and the Sichuan dataset (A2), we made our inferences more robust to potential clustering effects by constructing bootstrapped estimates of the coefficients based on fitting the model to 10,000 re-samplings of the data with replacement.

For all the FB-predictor models, we included data only from women interviewed who were born before 1943 (n = 7521) and fb *before* marriage (99.4 ± 0.2 percent of fb women born before 1943 were fb before marriage; *n* = 3841). (Table 8 shows the data restrictions on the models.) We limited the data this way in order to assess FB in women’s premarital households, where virtually all decisions about FB were made, and to assess female hand labor before the many changes implemented by the People’s Republic of China (PRC), changes that largely began in 1950 (see Appendix A and Appendix B).

**Table 8:** Data restrictions on the models.

restriction	Models A1 & A2	Models A7, A8 & A9	Model B1	Models C1 & C6
B (birth year < 1943)	yes	yes	yes	yes
F (if fb = 1, fb before marriage)	yes	no	no	yes
1 (if fb = 1, fb at least 1 year)	yes	no	no	yes
M (marriage year < 1950)	no	no	(yes)^a^	yes
x (exclude if spinAny = no)	no	no	no	yes

^a^ This restriction was not formally imposed, but it obtained by definition.

To assess whether our data allows us to consider whether girlhood knowledge of prohibitions correlate to unbinding, we conducted log-likelihood ratio (G) tests on three contingency tables (presented in Results below). For each, we calculated the expected values in each cell (under the null hypothesis) as the product of the marginal values. We examined data across BBG sites that contained the necessary data (model A7, *n* = 683), restricting the data only by birth year, and compared these results to the only two sites (both in Yunnan Province, see Fig 6) where extensive qualitative remarks make us confident that only *permanent* unbinding remains in the unbinding category (model A8 *n* = 35; model A9, *n* = 114).

### B. generational model (B1)

We assessed the hypothesis that FB and spinning were not independent for one previous generation, asking whether the fb mothers of women interviewed were more likely to spin than non-fb mothers (Table 7). We conducted a log-likelihood ratio (G) test on the contingency table and calculated the expected values in each cell as the product of the marginal totals. In this generational model, mothers of all women in the (BBG) dataset were necessarily born before 1943 (Table 8). However, there was no way to know whether mothers were bound before or after marriage.

### C. labor models (C1–C6)

We tested whether commercial production correlated with the spinning-labor contributions a woman had made to her premarital household in two ways. We asked separately whether commercial production correlated with the daily amount of cotton spun (the “daily-labor” models, C1–C5) and whether it correlated with the total number of years that a girl spun in her premarital household (the “labor-years” model, C6). We defined commercial spinning as spinning (usually in the girls’ own home) that produced thread sold in local markets (usually by girls’ relatives), thread commissioned for a wage (usually by another villager), or thread exchanged directly for other goods (usually for salt, cooking oil, rice, finished cloth, and/or raw cotton). In contrast, we defined domestic spinning as producing thread used within girls’ own homes (i.e., to weave cloth; weaving was similarly defined as commercial or domestic). Some girls produced thread for both commercial and domestic purposes (Tables 3–6). For these labor models, we contrasted girls who did *any* commercial production (including those who did only commercial production and those who did both commercial and domestic production) with girls who did *only* domestic production (no commercial production at all).

For all the labor models (C1–C6), we restricted the sample from the BBG dataset to include only women who were married before 1950 in order to avoid the many labor-related changes during the Maoist period (1950–1976). Under Maoism, commercial rural handicraft production was discouraged or banned outright [34]. Thus, daily hand labor was greatly reduced for girls and young women during the Maoist period; most women interviewed reported doing agricultural labor all day for work points and spinning, weaving, or doing other hand labor at night because they received no work points for their hand labor. Moreover, premarital labor-years increased for many women with implementation of the 1950 Marriage Law, requiring that all brides be at least 17. As with the FB-predictor models, we included only women who were fb before marriage (Table 8).

#### Daily-labor models (C1–C5)

We modeled the amount of cotton spun per day as the response variable in a multiple linear regression with commercial production as the hypothesized correlate. In model C1, we included all the following covariates: county, commercial and domestic spinning (yes/no), other hand-labor experience (yes/no), age learned to spin (in *sui*), education (some/none), and whether or not the natal family owned a loom (yes/no). Models C2–C5 examine variation at the county level; they use the same variables as above but exclude “county” as a variable. (These models are presented in S2 Analysis: County Results for the Daily-Labor Model [C2–C5]. The variables for these models are discussed in Appendix B.)

The distribution of the amount of cotton spun was right-skewed (Shapiro-Wilk test; *p*<0.001). Therefore, this variable was modeled with an exponential transformation, *x*′ = *x^λ^*, with *λ* optimized to maximize the *p* value in the Shapiro-Wilk test. Interaction terms were not considered due to insufficient degrees of freedom. As with the FB-predictor models A1 and A2, we increased the robusticity of our inferences in the multiple-county, daily-labor model (C1) by constructing bootstrapped estimates based on 10,000 re-samplings with replacement.

#### Labor-years model (C6)

To test the hypothesis that commercial spinners labored more years for their natal families than domestic spinners, we defined spinning labor-years as the difference between the age that a girl learned to spin and her age at marriage. The distribution of labor-years was significantly nonnormal (Shapiro-Wilk test; *p* < 0.001). Being unable to normalize the distribution, we used a Wilcoxon rank sum test with continuity correction to test for a difference in labor-years between commercial spinners and spinners who spun for domestic use only.

## Results

Our analyses suggest economic correlates to FB, going back at least one generation, and suggest a commercial benefit of girls’ production of handicrafts to their rural premarital households. We found repeated evidence of the importance of county-level variation and evidence of a decrease in FB over time.

### A. FB-predictor models (A1–A9)

In both the BBG and Sichuan datasets overall (models A1 and A2 respectively), all the following variables were significant: county, birth year, natal family wealth, girlhood commercial hand-labor experience, and some educational measure (Tables 9 and 10). Having heard of a prohibition against FB was *not* a significant predictor of FB status. Because models were reduced using iterative deletion and only the best-fitting models are presented, variables that were not significant—including the FB prohibition variable—dropped out of models A1, A3 –A5. We ran the best-fitting models again with the insertion of the FB prohibition variable in order to assess whether its *p*-values in any of the models was near significance. They were not (model A1; *p* = 0.957); nor did the addition of that variable alter the significance level of other variables. (Agricultural labor and domestic production results are discussed below.) The bootstrap estimates confirmed the significance of all variables except commercial hand-labor (discussed below). A woman was *less* likely to have been fb, the later her birth year (*p* < 0.001 for both A1 and A2). The subsamples (*n*) meeting all restrictions and providing all variables were 1485 for model A1 and 4567 for model A2.

**Table 9:** FB-predictor model A1 (BBG dataset; data restrictions: BF1).

Coefficient	Estimate	Std. Error	Z Value	Pr(>|Z|)	
Intercept	542.94	37.41	14.51	< 0.0001	***
County 1101	1.46	0.64	2.29	0.02	*
County 1102	1.33	0.66	2.01	0.04	*
County 1701+2	-3.42	0.84	-4.08	< 0.0001	***
County 1901	13.61	468.60	0.03	0.98	
County 1902	3.06	0.70	4.34	< 0.0001	***
County 2001	1.43	0.68	2.11	0.03	*
County 2002	1.36	0.60	2.28	0.02	*
County 2101	-2.08	0.75	-2.76	0.006	**
County 2102	-1.14	0.69	-1.65	0.10	.
County 2301	-0.31	0.63	-0.48	0.63	
County 2302	-1.84	0.66	-2.76	0.006	**
County 2701	0.46	0.66	0.71	0.48	
County 2801	1.09	0.59	1.84	0.07	.
County 2802	1.20	0.67	1.79	0.07	.
County 2902	-2.53	0.88	-2.86	0.004	**
County 2903	1.35	0.71	1.89	0.06	.
County 3102	4.99	0.74	6.77	< 0.0001	***
County 3103	2.06	0.58	3.55	0.0004	***
Birth Year	-0.28	0.02	-14.57	< 0.0001	***
Mother Any Education	-1.34	0.62	-2.15	0.03	*
Hand Labor–Domestic	0.48	0.25	1.90	0.06	.
Hand Labor–Commercial	0.48	0.19	2.49	0.01	*
Agricultural Labor	-0.43	0.23	-1.89	0.06	.
Mother Footbound	1.83	0.42	4.31	< 0.0001	***
Marriage Mobility Index	0.31	0.09	3.66	0.0002	***

R Sq: 0.66. Adj. R Sq: 0.65. Signif. codes: 0 ‘***’ 0.001 ‘**’ 0.01 ‘*’ 0.05 ‘.’ 0.1 ‘ ‘ 1

**Table 10:** FB-predictor model A2 (Sichuan dataset; data restrictions: BF1).

Coefficient	Estimate	Std. Error	Z Value	Pr(>|Z|)	
Intercept	354.28	14.89	23.80	< 0.0001	***
County DZ	0.49	0.16	3.11	0.002	**
County EM	0.70	0.17	4.23	< 0.0001	***
County JJ	-0.46	0.16	-2.98	0.003	**
County LQ	1.21	0.18	6.87	< 0.0001	***
County LZ	1.36	0.17	7.86	< 0.0001	***
County MS	-1.01	0.16	-6.36	< 0.0001	***
County NC	-0.06	0.15	-0.37	0.71	
County SN	0.24	0.16	1.53	0.13	
County ZG	-0.28	0.16	-1.73	0.08	.
Birth Year	-0.18	0.01	-23.82	< 0.0001	***
Ego Literacy	0.31	0.14	2.56	0.02	*
Hand Labor–Commercial	0.50	0.08	6.21	< 0.0001	***
Marriage Mobility Index	0.15	0.02	6.42	< 0.0001	***

R Sq: 0.33. Adj. R Sq: 0.32. Signif. codes: 0 ‘***’ 0.001 ‘**’ 0.01 ‘*’ 0.05 ‘.’ 0.1 ‘ ‘ 1

**Fig 11:**
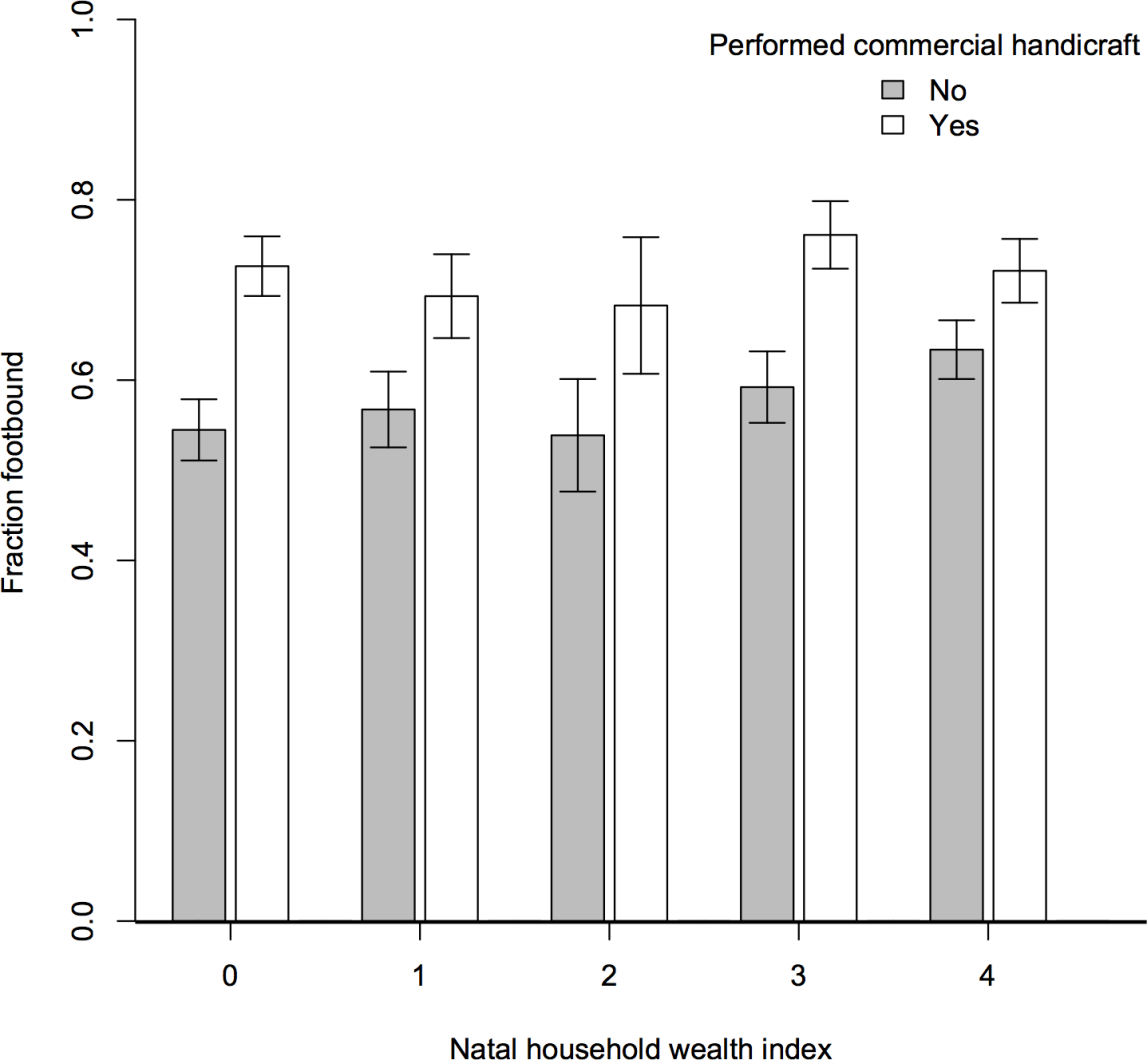
Fraction of women born 1887–1942 who were footbound in 20 counties in rural China by natal household wealth and commercial handicraft production (*n* = 5373; error bars indicate the 95% CI). Both natal household wealth and whether a girl performed commercial handicraft labor predicted the likelihood of the girl being footbound (logistic regression, both *p* < 0.001 when all data were pooled). The wealth index was scored as 2 points for family ownership of land, and 1 point each for ownership of a house or draft animal ([18], Appendix B).

A woman was *more* likely to have been fb if her natal family was wealthier (*p* = 0.014 for A1; *p* < 0.001 for A2) and if she had commercial hand labor experience (*p* = 0.020 for A1; *p* < 0.001 for A2). Doing any kind of handicraft production was a significant predictor of FB (Fig 11). However, as presented in S1 Analysis (which includes both explanatory text and Table A), there was regional variation in the importance of specific handicrafts and in commercial versus domestic production. In the Northern region, commercial spinning and domestic weaving were significant; in the Central region, domestic weaving; in the Southwest, other handicraft production (such as weaving reed baskets and mats); and in Sichuan, commercial spinning, domestic weaving, and other commercial handicraft production were all significant (see S1 Analysis). We think this regional variation explains why the bootstrap estimates showed commercial hand-labor experience no longer significant in the BBG dataset (model A1, 95% CI for the coefficient [-0.004, 0.856]). Bootstrapping showed commercial hand-labor experience remained highly significant in the Sichuan dataset (model A2, significant at the 99.9% CI), which sampled from only one region.

Which of several educational measures was significant differed between models A1 and A2, but both models showed a negative correlation between FB and education. In model A1 (BBG), women whose mothers had some education were less likely to be fb (*p* = 0.029, Table 9); in model A2 (Sichuan), illiterate girls were more likely to be fb than literate ones (*p* = 0.031, Table 10).

Model A1 (BBG) yielded three additional findings. Mothers who were fb were more likely to have daughters who were fb (*p* < 0.001). Women who produced handicrafts for *domestic* use (regardless of whether they also did commercial hand-labor) were more likely to have been fb (*p* = 0.041). Agricultural labor was *not* significant, but women with agricultural field labor experience (including planting, weeding, harvesting, and winnowing) were *less* likely to have been fb (*p* = 0.078).

The results of G tests on the prohibition and unbinding variables (models A7, A8, and A9) are contradictory. Looking across the BBG dataset, there *is* a significant relationship (A7, *p* = 0.017): those who were both unbound and who had heard of a prohibition, and those who were not unbound and had not heard of the prohibition, occur at greater frequencies than expected (Table 11). However, when we look only at the two Yunnan counties where we are confident that the data indicate *permanent*, not temporary, unbinding (A8, A9), then the results are *not* significant (Tables 12 and 13).

**Table 11:** Prohibitions and unbinding (BBG dataset; data restriction: B).

		Heard of prohibition		
		yes	no	total
unbound before 1949	yes	410 (60%)	153 (22.4%)	563 (82.4%)
	no	74 (10.8%)	46 (6.7%)	120 (17.6%)
	total	484 (70.9%)	199 (29.1%)	683 (100%)

Log likelihood ratio statistic (G) = 5.7271. X-squared df = 1. *p*-value = 0.0167

**Table 12:** Prohibitions and unbinding (Yunnan County 3102; data restriction: B).

		Heard of prohibition		
		yes	no	total
unbound before 1949	yes	9 (25.7%)	2 (5.7%)	11 (31.4%)
	no	18 (51.4%)	6 (17.1%)	24 (68.6%)
	total	27 (77.1%)	8 (22.9%)	35 (100%)

Log likelihood ratio statistic (G) = 0.205. X-squared df = 1. *p*-value = 0.6507

**Table 13:** Prohibitions and unbinding (Yunnan County 3103; data restriction: B).

		Heard of prohibition		
		yes	no	total
unbound before 1949	yes	66 (57.9%)	15 (13.2%)	81 (71.1%)
	no	22 (19.3%)	11 (9.6%)	33 (28.9%)
	total	88 (77.2%)	26 (22.8%)	114 (100%)

Log likelihood ratio statistic (G) = 2.7862. X-squared df = 1. *p*-value = 0.09508

### B. generational model (B1)

We found a significant relationship between FB and spinning for the mothers of women in our BBG dataset (Table 14, G = 5.3824; *p* = 0.020). There were more mothers who both spun and were FB, and more mothers who were not FB and did not spin, than expected under the null hypothesis. For a subsample of the BBG dataset, there was information on whether their mothers were fb (*n* = 2486) and spinners (*n* = 997; for both *n* = 956): 90.1 ± 1.2 percent of mothers were fb, and 74.8 ± 4.0 percent of mothers spun. We estimated mothers’ birth years as daughter’s birth year minus 25, resulting in a range of birth years from 1882 to 1931 for mothers with data on whether they were fb (see also Figs 7–10).

**Table 14:** Mothers’ FB and spinning (BBG dataset; data restriction: B).

		Mother spun		
		yes	no	total
Mother FB	yes	414 (43.3%)	460 (48.1%)	874 (91.4%)
	no	28 (2.9%)	54 (5.6%)	82 (8.6%)
	total	442 (46.2%)	514 (53.8%)	956 (100%)

Log likelihood ratio statistic (G) = 5.3824. X-squared df = 1. *p*-value = 0.02034

### C. labor models (C1–C6)

Doing any commercial spinning (regardless of whether they also did domestic spinning) *did* predict women’s spinning-labor contributions to premarital households before 1950.

#### Daily-labor models (C1–C5)

**Fig 12:**
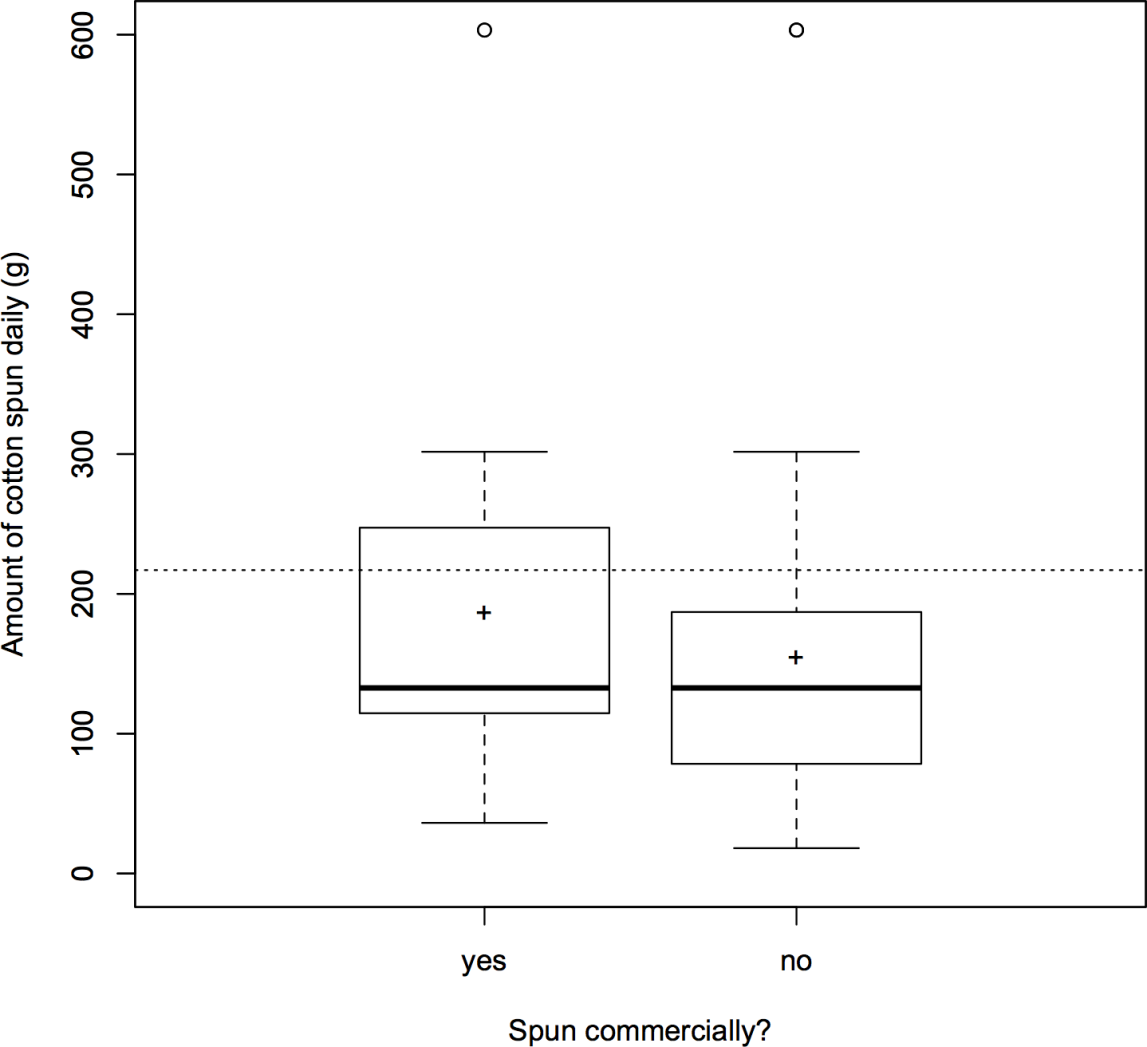
Amount of cotton spun daily by Chinese girls born 1907–1943 (*n* = 137), subdivided by whether they spun commercially, which was significant (multiple regression: *p* = 0.002) when other covariates were considered (model C1). Boxes indicate the interquartile range (IQR), and whiskers extend to the farthest point ≤ 1.5 times the IQR. Individual points beyond the whiskers are plotted as circles. Medians are marked by the horizontal bar, and means by “+”, for direct comparison with average spinning rates Thomas Jefferson reported for enslaved girls at his Monticello and Poplar Forest plantations, indicated by the dotted line [35–37].

In the multiple-county, daily-labor model (C1), both county and commercial spinning were significant (*p* < 0.05 and *p* = 0.002, respectively; Table 15). Notably, FB did *not* predict lesser daily production (contra [5, 7–8, 19]); neither did it predict greater daily production. The fitted model predicts that, at the median age of 12 *sui* (approximately 11 years old), commercial spinners spun 42 grams more per day than noncommercial spinners (Fig 12), although there was significant variation in the daily amount produced across counties (models C2–C5 examining county-level variation are presented in S2 Analysis). The BBG subsample (*n*) meeting all restrictions and providing all variables was 137 (see Appendix B). The bootstrapped estimate confirmed commercial spinning as a significant predictor (99% CI for the coefficient).

**Table 15:** Daily-labor model C1 (BBG dataset; data restrictions: BFM1).

Coefficient	Estimate	Std. Error	Z Value	Pr(>|Z|)	
Intercept	0.94	0.01	94.99	< 0.0001	***
County 1101	0.04	0.02	1.77	0.08	.
County 1102	0.03	0.01	2.05	0.04	*
County 1701+2	0.01	0.01	0.49	0.62	
County 2001	0.02	0.01	2.27	0.02	*
County 2002	0.01	0.01	0.38	0.70	
County 2101	-0.01	0.01	-0.77	0.44	
County 2102	0.01	0.01	1.19	0.24	
County 2301	0.01	0.01	0.49	0.63	
County 2302	0.01	0.01	0.69	0.49	
County 2902	0.005	0.01	0.46	0.65	
County 2903	0.01	0.01	0.46	0.65	
County 3103	-0.02	0.02	-0.95	0.35	
Spin–Commercial	0.01	0.004	2.76	0.007	**

R Sq: 0.22. Adj R Sq: 0.14. Signif. codes: 0 ‘***’ 0.001 ‘**’ 0.01 ‘*’ 0.05 ‘.’ 0.1 ‘ ‘ 1

#### Labor-years model (C6)

Commercial spinners labored more years before marriage than noncommercial spinners (*p* = 0.028; Table 16). The first quartile was 0.4 years longer for commercial spinners, the median was 0.5 years longer for commercial spinners, and the third quartile was 1 year longer for commercial spinners. Maximum values were also greater for commercial spinners. The BBG subsample (*n*) meeting all restrictions and providing all variables was 136 for commercial spinners (97 of whom also spun for domestic use) and 229 for spinners who spun for domestic use only.

**Table 16:** Daily-labor model C1 (BBG dataset; data restrictions: BFMx1).

		Labor Years Quantile Values				
		Min.	25%	Median	75%	Max.
Spun Commercially	Yes	0	3.9	6.5	9.0	19.0
	No	0	3.5	7.0	8.0	14.0

Wilcoxon rank sum test with continuity correction. data: spun commercially = ‘y’ and spun commercially = ‘n’. W = 17711, p-value = 0.02802. n1 = 136; n2 = 229

## Discussion

Our results indicate that the commonly held assumption that FB limited female economic contributions to households is wrong. Rather, handicraft production—both commercial and domestic—was a significant factor in FB for at least the last two generations of fb rural Chinese women in inland provinces. Natal families held onto girls with commercially valuable handicraft skills longer, suggesting that the economic contributions from their higher daily production were important to women’s premarital households.

Additionally, our evidence raises questions about assumptions that government prohibitions ended FB. Girlhood knowledge of FB prohibitions was irrelevant to whether women were ever fb or fb for at least one year of age, a result we take as an indirect indicator that FB prohibitions were not effective (S1 Analysis). One might wonder whether these prohibitions correlate to permanent unbinding. Although we did find a significant relation (*p* = 0.017) between the prohibition and unbinding variables across the BBG data (model A7, Table 11), we consider this result unreliable for the following reasons. Qualitative reports (for example, by interviewed women IDs 1702040, 1901041, 1902058, 2301100, 2702020, 3102010, 3102040, 3102069, 3103142) indicate that prohibitions—including associated fines—often led to *temporary* unbinding. In the two Yunnan counties (*n =* 149) where we are confident that only permanent unbinding is represented in the unbinding variable, we find no correlation between the prohibition and unbinding variables (A8–A9, Tables 12 and 13). We interpret these results taken together to mean that, in the bulk of the BBG dataset, the unbinding variable does *not* capture permanent unbinding. Rather, it suggests that girls who had heard of prohibitions were more likely to *temporarily* unbind (cf. [38]). These reports also mean that there are no reliable quantitative data to test whether FB prohibitions led to *permanent* unbinding in China before 1949.

All of the models (A1–A6) are very clear that a girl’s having heard of prohibitions in no way correlated with whether she was ever footbound or footbound for one year of age or longer. One might ask whether this indirect indicator that prohibitions had little effect on the binding of *rural* girls can speak to the demise of FB in China’s coastal areas and large cities. These rural, inland findings raise questions about the presumption that government prohibitions and campaigns by activists—Chinese and Western, missionary and secular—were responsible for the end of FB in coastal and urban areas. It appears that, during the first two decades of the twentieth century, people in coastal cities stopped binding their daughters’ feet (e.g., [5, 6, 8]), though there are no quantitative surveys to confirm the qualitative reports of the day. We do know that there were concerted early 20^th^-century political and religious efforts to eradicate FB (Appendix A), but we also know that during this same period there were myriad factories and mills springing up in coastal cities [39, 40]. They produced many items—especially thread and cloth—that had previously been hand-produced, and they were along routes of easy waterway transportation ([25, 39]; see also Fig 4). Our inland, rural findings suggest that, given the coincidence in China’s coastal cities of FB’s demise, FB prohibitions, *and* industrialization of previously hand-produced goods, we can no longer assume that prohibitions caused FB’s demise. Rather, FB’s correlation to handicraft production and not to prohibitions in rural areas suggests that future research should seek to investigate whether industrialization might not have been more important to the end of FB in coastal sites than previously credited.

The negative correlation in our data between birth year and FB status (models A1–A6) is not surprising, given that we know FB was ending as a custom beginning from the mid-19^th^ century ([5–6, 8–9, 18], Figs 7–10). Arguably, the significance of county-level differences (models A1–A6, C1) may be understood from the great variability historically documented in local-level transportation networks in early 20^th^-century China [23, 25, 29] in combination with the correlation we show of FB to commercial handicraft production (Figs 3–6 and 11, S1 Analysis, S2 Analysis). We are able to add to this combination qualitative reports by elderly men who had been medium- to long-distance traders before 1950 [23] and our findings of the *lack* of significance of girlhood knowledge of FB prohibitions. Taken together, these lines of evidence strongly suggest that FB ended when railroads, dredged waterways, or improved roads allowed transport of thread, cloth, and other goods produced in distant urban factories into rural, county markets so cheaply that these factory goods undersold rural home-produced commercial handicrafts. When raw cotton sold for more than homespun cotton thread did in a local rural market (as qualitatively reported, for example, by interviewed women, IDs 1701036 and 2702066; cf. [41–42], hand-spinning became worthless. This economic explanation fits the mosaic pattern of FB’s demise across rural China.

Whether a woman’s mother was fb had a lot of predictive power in the BBG dataset (model A1), which sampled 20 counties across 11 inland provinces. It made a larger contribution to FB than one year’s difference in birth, one unit of change in our rural wealth index, or commercial hand-labor experience. Since this predictor was not available in the Sichuan dataset, we are somewhat less confident in the results for Sichuan (model A2). The significance of a woman’s mother being fb might be due to vertical cultural transmission of a practice based on a belief (cf. [43], but see [11–12, 44]). However, such interpretation is complicated by the necessity of knowing *how* to bind feet [45] in order to transmit the custom: some women explained never having been bound because of their mother’s absence from death or remarriage or having to stop binding because their mother’s absence meant the binding was not done properly (qualitative reports: IDs 1102004, 1501131, 2002090, 2102173, 2301008, 2302007, 3102015, 3103102, 3103131). FB also had health risks: 24.3 ± 1.3 percent of fb women (*n* = 4151) reported their feet became infected. Because the women interviewed survived to be included in our study, we cannot know whether the presence of a fb mother reduced a girl’s risk of death due to infection from FB.

We find a complex relation between wealth and FB (models A1 and A2; Fig 11). Families ranked higher on our *rural* wealth index were more likely to bind daughters’ feet. Handicraft production required initial capital (qualitative reports: IDs 1901021, 2702076, 2902050, 3102012; cf. [7, 14, 41–42, 46]). Spinning required raw cotton (or other fiber) and a spinning wheel; weaving cloth required thread, fuel and water to boil and soften the thread, and a loom; weaving mats required reeds; etc. Some women rented a loom or wheel for a portion of the product, but that required finding a wheel or loom not in use. Some households could not afford to purchase or produce even footbinding cloth (qualitative reports: IDs 1101010, 1501023, 2001004, 2701031, 2801128, 3102036). We suggest that rural families needed sufficient capital to fund handicraft production in order to encourage FB.

However, a different type of wealth indicator—female education/literacy—*decreased* the likelihood of FB. We suggest this difference can be understood because female education indicated not only greater wealth than our rural wealth index captures but also urban wealth. Female education was more likely in cities [47–48]. Circa 1930, Buck estimated sufficient literacy to read a letter in rural China at 30 percent for men and only 1 percent for women ([7], cf. Table 1). FB thus appears inversely correlated to the highest socioeconomic class—only the top few percent in rural areas—and to urban wealth. It may be that other processes influenced the demise of FB among the small percentage of women in China’s highest socioeconomic strata—processes such as gender identity [5], pursuit of “modern” lifestyles [2, 5, 6, 9], or perhaps even prohibitions [2, 5, 9]. However, given evidence from premodern China that elite women were involved in household handicraft production to a significant degree [14, 46], we caution against the assumption that economic factors were unimportant for the end of FB among China’s elite women, and we suggest that this assumption warrants future research.

The most important results of our study show FB was *not* an economically disinterested custom in which families gave up daughters’ labor to promote their marriageability. FB has been portrayed as economically disinterested [1–8] because it was assumed to severely limit female labor. Although families usually received a “body price” (*shenjia* 身價), or brideprice (*pinjin* 聘金), at a daughter’s marriage, it was not perceived as offsetting the financial burden of raising a daughter [49]. Moreover, most fb women did not marry to wealthier families [18], so there is no reason to expect families received greater brideprices for fb brides than never-bound brides.

Using median values for birth year and household wealth, and median coefficients for categorical values, the fitted FB-predictor models (A1 and A2) indicate that girls producing commercial handicraft were 1.24 times more likely to have been footbound than girls not producing commercial handicraft across China (model A1, BBG dataset), and 1.05 times more likely in Sichuan (model A2) (contra [38]). Footbound girls’ hand-labor earned cash and goods for their premarital households. Furthermore, the significant correlation between mothers’ FB status and mothers’ labor as spinners (generational model, B1) corroborates evidence from the Sichuan regional FB-predictor model (A6, see S1 Analysis) that the economic contributions of fb women go back at least to the 1880s.

Initial comparison to early 19^th^-century rural US spinning shows that assessment of Chinese daughters’ economic contributions to their households before marriage is complex. Based on the fitted values of the daily-labor model (C1), Chinese commercial spinners who learned to spin at the median age (about 11 years old) produced 1.4 times as much per day as girls with no commercial spinning experience: 139 versus 96 grams (Fig 12). Daily labor predictors and rates varied by county (models C2–C5), and FB apparently did boost production in some locales (model C3; Tables A and B in S2 Analysis). In some counties, Chinese girls produced more than the 217 grams per day that was the average daily amount spun by African American girls enslaved at Thomas Jefferson’s Monticello and Poplar Forest plantations (circa 1800) using comparable technology [35–37]. That Chinese girls could produce as much or more per day as enslaved girls with self-interested overseers suggests that Chinese daughters’ economic contributions to their natal families were potentially substantial (see Appendix C: Comparing Labor Coercion via Slavery and Footbinding).

FB has been used [2, 9] as a model for ending female genital cutting and honor killings because all these customs have been assumed to have no economic correlation. Evidence presented here that families had economic interests for FB and indirect evidence that government prohibitions against FB had no significant effect on whether girls were fb suggests a need to re-evaluate whether economic interest may also influence these other customs.

## Appendix A: Historical context

FB probably began as a custom no earlier than the 10^th^ century [5, 20, 46] and ended at different times in different parts of China between the mid-19^th^ and mid-20^th^ centuries [18, 23]. There were many efforts to end FB. For example, Qing-dynasty official and reformer Kang Youwei in conjunction with Liang Qichao and others persuaded the Guangxu emperor to officially ban FB in 1898, arguing that FB shamed China before the West and handicapped China in the competition of nations by removing or reducing women’s economic contributions. The prohibition was soon rescinded but reinstated in 1905. The reformer Liang Qichao was so influential that his “image of women with bound feet as parasites, beasts, and slaves [became] the standard view” [5]. Christian missionaries from the US and Europe banned their converts and students from FB, and Mrs. Archibald Little founded the Natural Foot Society in 1898. During the Republican period (1911–1949), FB was banned repeatedly by different regimes, including the Nationalist government and warlords Yan Xishan (who ruled Shanxi Province) and Feng Yuxiang (who controlled Hebei, Henan, and parts of adjacent provinces during the 1920s and early 1930s). All these authorities sent soldiers to suppress FB and/or inspectors to levy fines on families with footbound girls or women [17, 23, 42]. The Chinese Communist Party discouraged FB in areas under their influence, though warfare (resistance to the Japanese invasion and occupation as well as the civil war against the Nationalists) limited their anti-FB efforts before the 1949 founding of the PRC. Today FB exists only among a rapidly diminishing number of elderly Chinese women.

Handicraft production, especially of textiles, continued in rural China for decades beyond the industrialization of textile production in China’s urban centers [7, 17, 23, 34, 42, 50]. Between 1900 and 1950, many parts of China experienced rapid expansion of transportation lines in the form of railroads, rivers, or dredged waterways capable of carrying large cargo ships and paved or packed-dirt roads capable of carrying cargo trucks. Such transport routes gave easy access to industrialized urban centers, where cloth-producing and thread-producing textile mills sprang up during the early 20^th^ century ([7, 23, 25–27, 51–52], Figs 3–6). Most of our research sites, however, were sufficiently removed from such transport routes that mechanized technologies for handicrafts (such as multi-spindle spinning jennies and iron-loom frames) were rare. For example, during the 1930s and 1940s, county 2001 was a two-day walk from the major urban center of Wuhan, yet women there reported using single-spindle spinning wheels (Figs A and B in S1 Images) and wood-frame looms (Figs C–F in S1 Images) at least through 1949 (and often much later). Thus, at the township and county level, transport conditions to and from markets affected the demand for homespun thread and home-woven cloth ([7, 23, 42], Figs G–J in S1 Images).

Home-based textile production was affected by the availability of raw materials. During the 1930s, cotton production fluctuated not only due to climatic influences but also due to policy impacts (for example, cultivation of opium competed with cotton in Shaanxi) and war [51, 53]. Some women in areas where cotton was not grown—especially Yunnan Province (counties 3102, 3103)—reported buying raw cotton to spin. Some also reported scavenging thread from old clothing to re-spin and re-weave into cloth (e.g., ID 3103201). In 15 of the 20 BBG counties, cotton was such a common crop that 35 percent or more of women interviewed reported their premarital families growing cotton (Table A in S2 Analysis; data on crops grown were not collected in Sichuan). In 13 of those 15 counties, at least 45 percent of households had unmarried daughters picking cotton. In these counties, cotton was readily available for local spinners to use.

## Appendix B: Detailed materials and methods

Ethics approval for use of the previously collected Sichuan data and for collection of the BBG data, including use of oral consent by human subjects, was granted by IRB no. 349 (panel 2) at Stanford University (protocol no. 83622, 2006–2011).

### Sites and sampling

Interview sites are distributed in inland rural China (see Figs 3–6). There are no sites in China’s southeast, coastal, or major cities because footbinding (FB) stopped so much earlier in these locales that we could not expect to find many living women who had experienced FB. Each “site” includes several natural villages, within a single township *(zhen* 镇, *xiang* 乡) when it was possible, but certainly within the administration (at the time of interviewing) of a single county, small city, or rural district of a large city (*xian* 县, *shi* 市, *qu* 区). Sites are referred to as “counties” in the main text. At every BBG site (except two), approximately 100–200 women were interviewed. In each natural village, we requested interviews with *all* mentally capable women living in the village who were old enough that some women of their generation had experienced FB; most women agreed to be interviewed. (Sampling bias thus includes survivorship and continued rural residence.) We included as many natural villages as necessary to interview 100–200 women. At site 2802, in Shandong Province, 55 women were interviewed. At site 1702 in Hebei Province, 63 women were interviewed, but since this site is within the same county as site 1701, sites 1701 and 1702 were pooled as a single site in the analyses. For site 1501, in Guizhou Province, data from all interviews with widespread internal inconsistencies and interviews without sufficient qualitative comments to verify internal consistency were removed, leaving a total of 94 women’s interviews included in the final BBG dataset. At each Sichuan site, 490–500 women were interviewed.

### Regions

We classified sites into regions based on G. W. Skinner’s well-accepted definitions of China’s historic macroregions ([24, 54–55] but see [56]). Our Northern region combines 8 sites in Skinner’s North region and 3 sites in Skinner’s Northwest region (Fig 4), with a total of 1080 women born before 1943 [18, 23, 51, 53]. All 6 sites in the Central region fall within Skinner’s Middle Yangzi region (Fig 5) and contribute a total of 1062 women born before 1943 [18, 23]. Although today Sichuan, Yunnan, and Guizhou provinces as well as the provincial-level city of Chongqing are considered part of China’s multi-ethnic southwest, we follow Skinner in dividing them into two regions: our Southwest region (Skinner’s Yungui region; Fig 6), totaling 404 women born before 1943, and our Sichuan region (Skinner’s Upper Yangzi; Fig 3), totaling 4973 women, all born before 1930 (cf. [18, 22, 23, 41, 42]). All sites in both these regions are ethnically Han (i.e., China’s ethnic majority, considered “ethnic Chinese” elsewhere in the world).

The maps (Figs 3–6) were constructed from historical maps of China available in Harvard University collections and online [24–29]. All historical base maps were geo-referenced and digitized by Harvard’s Center for Geographic Analysis and are archived there for scholarly use.

### Data-generating interviews

The interviews covered a wide range of questions for each woman’s natal household. Most women had vivid memories of their natal family from the time just before they left in marriage or just before the 1949 founding of the PRC (if they married later than that). However, not all women answered every interview question. “I don’t know” and “I don’t remember” were acceptable responses, and some questions depended on others. For example, who bound a woman’s feet could only be asked of women who had once been footbound (fb). Consequently, the number of data points differs across the models and descriptive statistics (Tables 1 and 2). The BBG interviews gathered some information not included in the Sichuan interviews, indicated below by “BBG only.” For example, the labor-years model (C6) uses the BBG data because the Sichuan data does not include the age at which women learned to spin.

Because the BBG survey spanned five years and these interviews represented the only possibility of gathering information on FB in relation to labor from women who experienced FB, some questions were added at later sites based on preliminary analyses. The generational and daily-labor models (B1 and C1–C5) use such added questions. The generational model (B1) uses data from women in 5 counties (2002, 2102, 2301, 2302, 3103) systematically asked who spun in their natal household, which allowed us to identify mothers who spun; women in another 5 counties (1501, 2001, 2902, 2903, 3102) occasionally volunteered this information, for a total of 956 women who provided data. The daily-labor models (C1–C5) use data from women in 8 counties (1501, 2001, 2002, 2101, 2102, 2301, 2302, 3103) systematically asked to recall the amount of cotton thread that they regularly spun per day; women in 8 other counties (1101, 1102, 1701+1702, 1901, 1902, 2902, 2903, 3102) occasionally volunteered this information, for a total of 463 spinners (born before 1943) who provided data. However, only 137 women fit all the restrictions and had all the necessary variables to be included in the multiple-county daily-labor model, C1. As far as we are aware, these are the only existing data on daily amount of cotton spun by individual Chinese handicraft producers before 1950. (Quantities were usually given in terms of “big ounces” [*liang* 两, 37.7 grams] or “catties” [*shijin* 市斤, 603.277 grams], but sometimes “spindles” or other local measures [*ding* 锭, *tuan* 团, etc.]. Only those quantities that could be converted to grams were used.) Because these models included only a subset of the data from the Central and Southwest regions, we recognize that the results may not generalize to the Northern and Sichuan regions.

We examine FB in relation to women’s premarital work and natal family conditions because almost all women were fb before marriage. Thus, we focus on the household in which adults decided whether to bind a girl’s feet. This focus also keeps the households under consideration in our study comparable, for we thus focus on households existing prior to the restructuring of rural labor following the 1949 founding of the PRC. By contrast, the postmarital households of women in our datasets include households existing both pre- and post-1949.

Our models use the following information collected: birth year; whether married before 1950; education and literacy levels of the woman interviewed, her mother, and her father; a natal-family wealth index; whether the woman herself had spun cotton or other materials (including spinning hemp or ramie, or reeling silk) before marriage, and if so, whether for domestic or commercial use; the age at which a woman learned to spin (BBG only); whether the woman’s natal family had a loom (BBG only); whether the woman had woven any fiber into cloth (including cotton, hemp, ramie, or silk) before marriage, and if so, whether for domestic or commercial use; whether each woman did any other premarital handicraft work, and if so, whether for domestic or commercial use. We explain these variables below.

### Women’s ages (variable: Birthyr)

Chinese traditional reckoning of years and ages required adjustment to international standards (based on Western reckoning). Women’s birth years were told to them most often in terms of the 12-year, lunar-based zodiac cycle. Moreover, Chinese traditionally reckoned age as 1 *sui* (岁) at birth with age added at the lunar new year. Thus, ages given in *sui* differ from the Western calculation of age (0 at birth, accruing age on one’s birth date) usually by 1 but sometimes by 2. Models use ages given in *sui*; the approximate Western age equivalent is noted in the text. In order to calculate precise birth years, women interviewed were asked both their age in *sui* at the time of the interview and their zodiac animal (*shu* 属).

### Data restrictions by birth year and marriage year

We restricted all models to data from women interviewed who were born before 1943 (see Table 8 for a summary of data restrictions) for the following reason. We looked at the median and interquartile range of ages at which FB first occurred for women in “majority” cohorts: 5-year birth cohorts in which 50 percent or more of the women were *ever* footbound [18]. The median age was 6.3 *sui* ± 0.1 (about 5 years old) and the 75th percentile was 8.0 *sui*, (about 7 years old; *n* = 3439). By restricting models to women born before 1943, we ensured that the youngest women (born 1942) would have an age within the 75^th^ percentile for FB by 1949, when the founding of the PRC dramatically changed the political economy and FB. This restriction removed 0 women from the Sichuan dataset and 189 women from the BBG dataset, leaving a total of 7521 women for consideration in the models. (This restriction was not necessary for the generational model B1 because mothers of women interviewed were necessarily born before 1943. However, because we removed data for women born later than 1942 in the final dataset, this restriction was effectively imposed in model B1.)

As explained in the main text, the labor models (C1–C6) were additionally restricted to women who married before 1950, in order to avoid Maoist-period influence. Because the generational model (B1) included the mothers of women interviewed, they were necessarily married before 1950. The FB-predictor models (A1–A9), however, did *not* include this marital restriction because of the extreme loss of data, once all the variables were included. Moreover, because these models examined whether *any* hand-labor was done, not quantity produced, they were less affected by Maoist-period influence. Girls and women still produced homespun cloth and other handicrafts throughout the Maoist period, though they were not able to do as much (given their increased agricultural labor); there was also a black market during the Maoist period where rural people sold or exchanged goods privately, especially textiles [34, 50, 51].

### Education (variables: EgoLit, egoAnyEd, mothEdAny, fathEdAny)

Women, their mothers, and their fathers were defined as having some education if reported to have *any* education prior to 1950 or, for mothers and fathers, if reported half- or fully literate. To read a Chinese newspaper with ease requires knowledge of 2000 to 3000 Chinese characters, to read a letter, about 1200 characters; before 1950, the ability to read a letter counted as literacy in rural China [7, 47–48]. People were defined as half-literate if they could read a little, for example, enough to understand a business sign.

### Rural wealth index (variable: MMI)

The rural wealth index used has an integer value between 0 and 4, depending on whether the woman’s natal family *owned*: any land at all (+2), a house (+1), or a draft animal (+1). Explained in more detail elsewhere as a “marriage mobility index” [18], the weighting in this rural wealth index is based on the primary importance to rural households of owning land (as opposed to renting it). It also recognizes that a house, which could serve as a workshop or business, or a draft animal, which could transport produce or goods, contributed significantly to rural household income.

### Production for domestic versus commercial use (variables: HandLabUse, handLabCom)

Domestic use was defined as consumption within the household, for example, cloth used for clothing worn by family members. Commercial use was defined as sale, wage, or direct exchange between households. Handicraft production for direct exchange between households was underreported in the interviews, because some women interviewed perceived such exchanges between households as domestic use (e.g., county 1101).

### Types of labor (variables: HandLab, anyAgLab, spAny, weAny, AnyHC, weHC)

Production tasks were classified as hand labor if they fell into the predefined categories of spinning, weaving, embroidery, making shoes, making clothes, making bedding, sorting tea, raising silkworms, or processing opium. There was also an open-ended “other” handicraft category of any additional labor task that women themselves defined as handwork. In our models, we focus on only three distinct categories of hand labor: spinning, weaving (cloth only), and other. The “other hand labor” category in the models includes, not only tasks predefined as hand labor, but also the other tasks that women themselves volunteered. In addition to the examples listed above, this “other” category includes twisting fibers into rope; weaving of fishnets, mosquito nets, reed baskets, and reed mats; and sewing, twisting grass (for fuel), and carding cotton.

Our agricultural field labor category includes only crop labor: planting, plowing, weeding, pruning, harvesting, hauling crops from the field, threshing, flailing, drying, winnowing, fertilizing, and watering. This category did *not* include other farm-labor tasks that girls and women did—most commonly, herding, and raising pigs or chickens. (Other types of nonagricultural labor that women commonly reported were collecting firewood or wild plant foods, housework, cooking, and childcare.)

### Footbinding (variables: EgoFB, fb1yr, mothFB, prohib)

The BBG and Sichuan datasets are the only known quantitative sources of the following information used here: whether a woman herself was ever footbound (fb), even briefly; if so, who bound her feet, and the duration of (first) binding; whether she herself had heard of FB prohibitions as a girl (BBG only); and whether the woman herself knew from her own experience that her mother was fb (BBG only).

Variation existed in the FB process, the size of feet produced, and whether it was possible to stop binding. To achieve the most extreme and much-lauded “lotus” (*lian* 莲) form (with feet approximately 10 cm long) was a painful process that required bending the toes under the foot and toward the heel, forcing the arch upward, and tightly binding with a cloth [6, 8, 57]. Over time, FB led to pressure breaks and sometimes the loss of toes (Fig 1, Fig A in S2 Images); women whose arch had been broken could never be completely unbound or they could not walk [8, 58]. A less extreme FB process forced the small toes under the sole of the foot, breaking the toes but not the arch [18]. Many women with this “cucumber” (*huanggua* 黄瓜) or “half-sloping” (*banpo* 半坡) form experienced FB as a phase of their lives, because as long as the bones of the arch were intact, let-out feet (Figs B–C in S2 Images) would—after a painful transition period—return to a form that functioned much as never-bound feet [8, 18].

We classified a woman as ever-fb if she was bound, even for one day: 57.3 percent (± 1.1, 95% CI) of women born before 1943 were ever fb. In assessing the percentage of Chinese women in our sample ever fb (Figs 7–10), the approximate 95-percent confidence intervals for proportion footbound ($\hat{p}$) in each region were calculated as observed proportion, *p*_*smooth*_, ± 1.96 (*SE*_*smooth*_), clipped to the range [0, 1], where *p*_*smooth*_ uses a Laplacian smoother of 0.5 to allow for estimates when observed proportions were 0 or 1:

$$p_{smooth}=\frac{n_{footbound}+0.5}{n_{total}+1},\;\mathrm{and}$$

$${SE}_{smooth}=\sqrt{\frac{p_{smooth}(1-p_{smooth})}{n+1}}.$$

When we added the restriction of considering only women married before 1950 (as well as born before 1943), the percentage of ever-fb women increased to 66.0 (± 0.1).

We initially ran the FB-predictor models (A1 and A2, including the regional models, A3–A6) as well as the labor models (C1–C6) using ever-fb women. However, because most ever-fb women reported unbinding their feet, at least for a time, we ran the models again with women whose footbinding duration was at least a year of age (by the lunar calendar). As stated in the main text, for models where data across all available sites were included (A1, A2, C1, C6), using all ever-fb women and using the year-plus FB restriction resulted in largely the same significant correlates (at *α* = 0.05; Table A in S1 Analysis). However, at the regional level (models A3–A6) and county level (C2–C5), there were interesting differences (discussed in S1 Analysis and S2 Analysis, respectively). We report models with the year-plus restriction in the main text because they systematically obtained a better fit (higher adjusted *r*^2^). Only in the daily-labor models at the individual county level where the low numbers were a factor (C3, C5), did using all ever-fb women (i.e., no restriction on the duration of being footbound) obtain a better fit (Tables A and B in S2 Analysis).

As discussed in the main text, although we sought to know whether and when women permanently unbound their feet, analysis of the unbinding data showed the responses to be problematic. Many qualitative reports of unbinding refer to temporary unbinding—that is, removal of the binding cloth, allowing the feet to “let out,” and then later rebinding (e.g., ID 3102017). Women’s remarks sometimes indicated resistance to FB by surreptitiously unbinding their feet at night only to be beaten and rebound the next day (e.g., ID 2903030; see also [18]). Remarks also indicated temporary unbinding during a lengthy illness or in anticipation of a visit from pre-1949 FB inspectors (e.g., IDs 2001046, 3102041) or for fear of the advancing Japanese army (e.g., ID 2301039). Given the conflation of temporary and permanent unbinding in women’s reports, we could not accept data in the survey category of “age of permanent unbinding” at face value. We thus compared findings on the relationship between unbinding and girlhood knowledge of the prohibition across regions (using all available data in the BBG dataset, model A7) with findings for two sites (3102, 3103), in Yunnan, where we are confident that the unbind variable includes only permanent unbinding (models A8 and A9).

We created a binary (yes/no) variable indicating whether a woman was fb a year or more before any kind of unbinding took place. This variable is inconsistent. As described above, ages were generally reported in *sui*, and the conversion to years is approximate. Additionally, people “are” an age for a year, so if binding was at the end of one age and unbinding at the beginning of the next age, actual time bound would be less than a full year. Nevertheless, this variable was given a “yes” value if the woman reported being bound for the duration of at least one *sui*. Thus, we eliminated women (*n* = 404) from consideration who bound very briefly in order to improve our assessment of long-term FB status.

We asked women whether they had heard of FB prohibitions as girls (before marriage) and before the founding of the PRC, when the government finally had the power to fully implement a FB ban (cf. [23]). (Note that FB rates did begin to decrease in our sites before 1949; see Figs 7–10 in the main text). By 2006, it was only possible to interview women subjected to FB (in the BBG sample); it was not possible to ask the older generation(s) who had decided whether to bind the feet of these women as girls. (In the Sichuan sample, women were not asked about prohibitions or about deciding to bind their daughters’ feet.) Thus, we can only indirectly assess the efficacy of FB prohibitions. We reason that, if government prohibitions effectively prevented families from binding girls’ feet, then those never-bound girls would be more likely to have heard of the prohibition—and remember the prohibition, since it helped them avoid such a painful process—than girls whose feet were bound. Women’s knowledge of FB prohibitions during their girlhood is therefore used as an indirect indicator of the efficacy of pre-1949 prohibitions against FB.

### Daily quantity of cotton spun (variable: SpCotAmt)

Women who spun cotton were asked how much they spun per day for their natal household. Because women were asked to recall the circumstances of their natal households just before they left it (usually in marriage, sometimes in adoption), these quantities generally represent the daily quantity produced by accomplished spinners.

## Appendix C: Comparing labor coercion via slavery and footbinding

Although the county-level results (presented in S2 Analysis) are certainly affected by small numbers, they nevertheless show the wide range of local variation, hence the persistent significance of county as a correlate across models. With regard to footbinding (FB), these results show that FB could serve as a form of labor coercion (in county 2102, model C3) but did not necessarily serve that function everywhere. Nevertheless, it is highly suggestive that the greatest estimated daily production values anywhere in our study come from footbound (fb) and uneducated girls in county 2102. Moreover, their estimated daily production values (252, 260, 320 grams) are considerably larger than the estimated amount from enslaved African American girls (217 grams [35–36]) or than the estimated amount from Chinese girls (undistinguished with regard to FB) during the early 1930s in a cotton-growing county across the river from our county 2702 (200 grams [51, 53]).

Comparative evaluation of FB as labor coercion is possible because Thomas Jefferson recorded textile production, including spinning, for his Monticello and Poplar Forest plantations. Between 1790 and the summer of 1812, when spinners were using a single-spindle wheel, Jefferson estimated that enslaved girls, aged 10–16 years old, spun an average of 7.67 ounces (217 g) of cotton per day, with work varying from 9 to 14 hours per day, depending on available sunlight [35–37]. Enslaved spinners were immediately supervised by older enslaved women, who spun or wove in addition to their other responsibilities; spinners had periodic oversight by Jefferson’s daughters, granddaughters, granddaughters-in-law, or an overseer’s wife, and they appear to have had daily quotas, possibly measured by “giving each [spinner] a certain weight of fiber first thing in the morning” [35]. Enslaved girls who failed to produce high enough quality textiles were threatened with shifting to full-time agricultural labor [35]. Oversight was intended to maximize labor outputs, approaching humanly possible maximums given the available technology. That the greatest daily production estimates for Chinese girls came from county 2102, where FB and lack of female education were significant correlates of the amount of cotton spun per day, is highly suggestive of FB as a form of labor coercion.

## Supporting information

S1 Images Spinning, weaving & cloth images. Figs A–J.
(PDF)

S2 Images Variation in bound feet. Figs A–C.
(PDF)

S1 Analysis Regional results for the FB-predictor model (A3–A6). Tables A–B.
(PDF)

S2 Analysis County results for the daily-labor model (C2–C5). Tables A–B.
(PDF)

S1 Dataset Relevant, anonymized BBG data. (XLSX)
