# Supplementary material for: Economic correlates of footbinding: Implications for the importance of Chinese daughters’ labor
Source: PLoS One. 2018 Sep 20;13(9):e0201337. doi: 10.1371/journal.pone.0201337 (PMC6147408; doi:10.1371/journal.pone.0201337)
Supplement: S1 Images — Figs A–J. (PDF) [file pone.0201337.s001.pdf]

S1 Images: Spinning, Weaving & Cloth

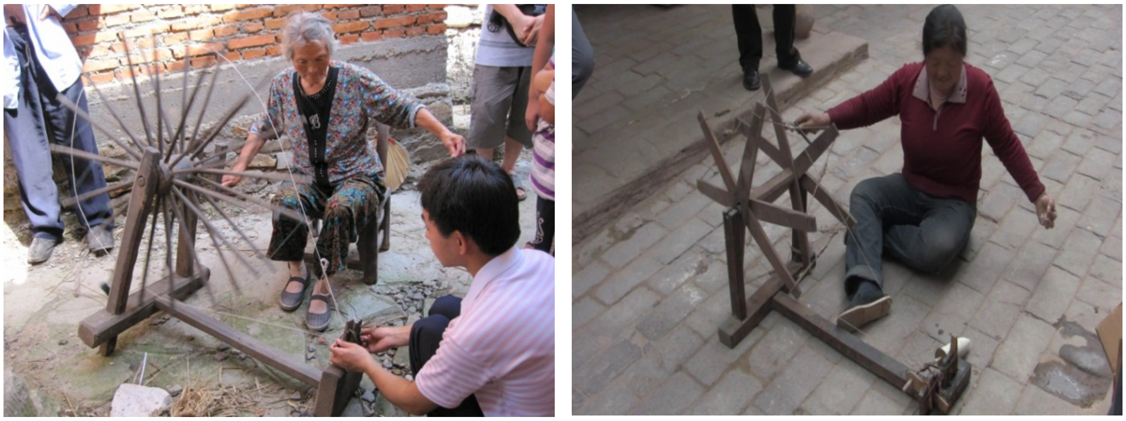

A. **B.**

Figs A and B. Women demonstrating use of single-spindle wooden spinning wheels. The larger type of wheel (**A**) was commonly used at research site 2001, photographed in July 2009, and the smaller type was commonly used (**B**) at research site 2902, photographed in April 2011. Women at site 2001 reported that girls had to be about 12 years old, which they considered older than usual, for their arms to be long enough to spin on such a large wheel.

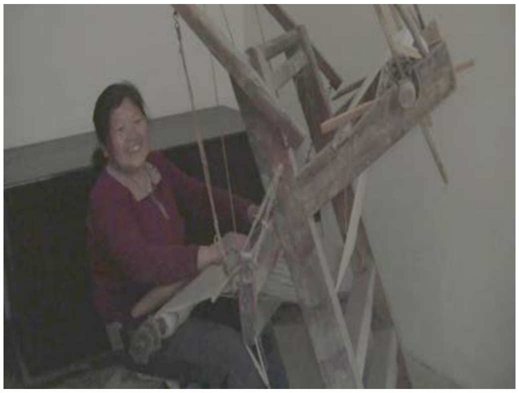

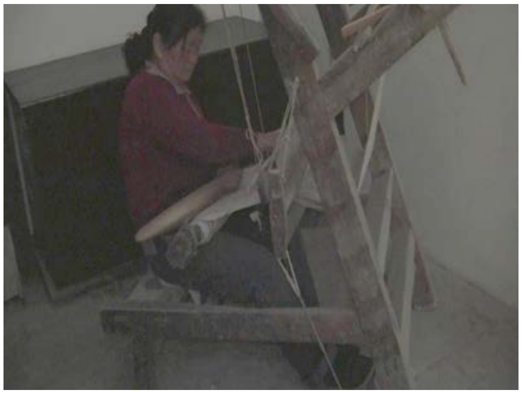

C. **D.**

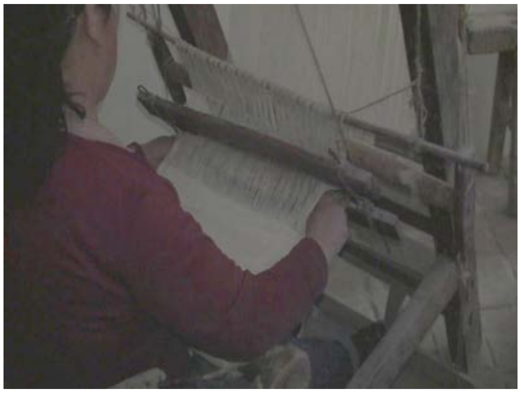

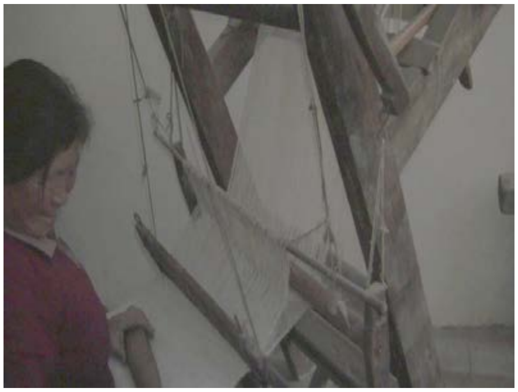

E. **F.**

Figs C, D, E, and F. Woman demonstrating use of a wood-framed loom. This type of loom was commonly used at research site 2902. Photographed in April 2011, these images show (**C**) the loom overall, (**D**) the reach needed to work the foot pedals, (**E**) pushing the shuttle, which carries the weft thread, through the warp threads, and (**F**) the warp threads strung on the loom frame.

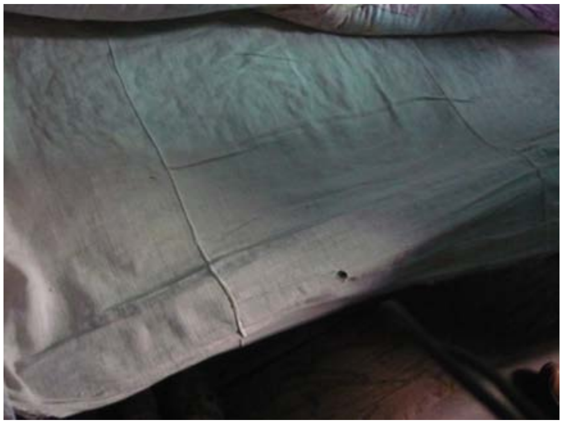
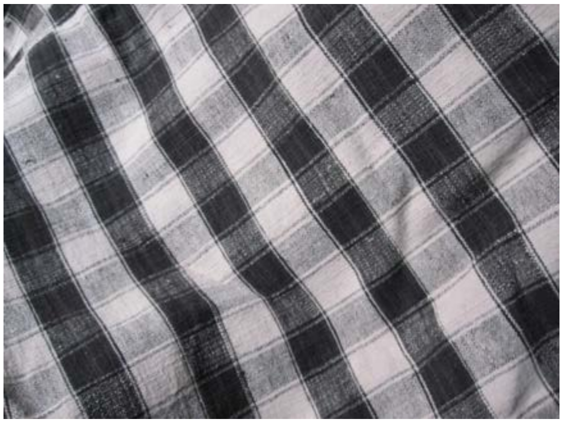

G. **H.**

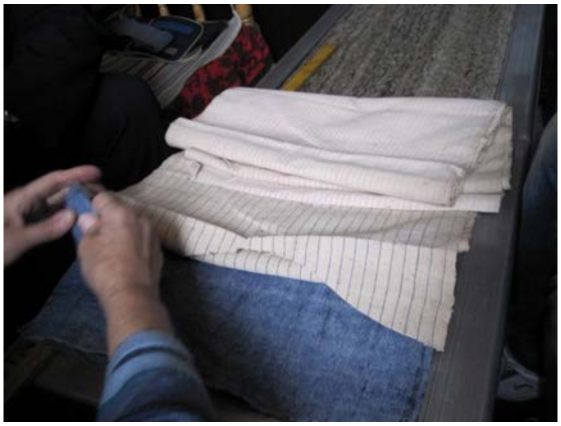
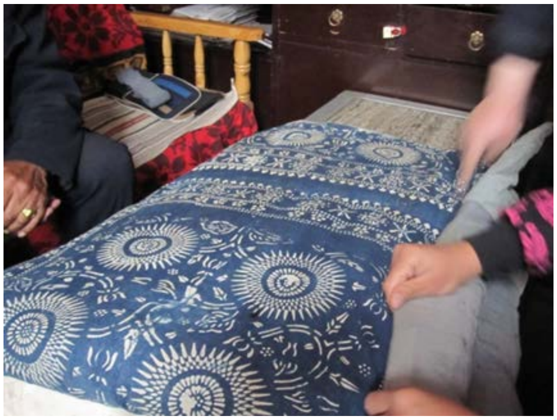

I. **J.**

Figs G, H, I, and J. Examples of homespun, home-woven cloth. These images, photographed in April 2011, are from research site 2101 (**G– H**) and research site 2902 (**I– J**). The cloth shown in **G** was reportedly woven decades previously and was in use as a bottom sheet. The remaining cloths (**H**, **I**, and **J**) were woven more recently and not in daily use.
