# Supplementary material for: Economic correlates of footbinding: Implications for the importance of Chinese daughters’ labor
Source: PLoS One. 2018 Sep 20;13(9):e0201337. doi: 10.1371/journal.pone.0201337 (PMC6147408; doi:10.1371/journal.pone.0201337)
Supplement: S2 Images — Figs A–C. (PDF) [file pone.0201337.s002.pdf]

S2 Images: Variation in Bound Feet

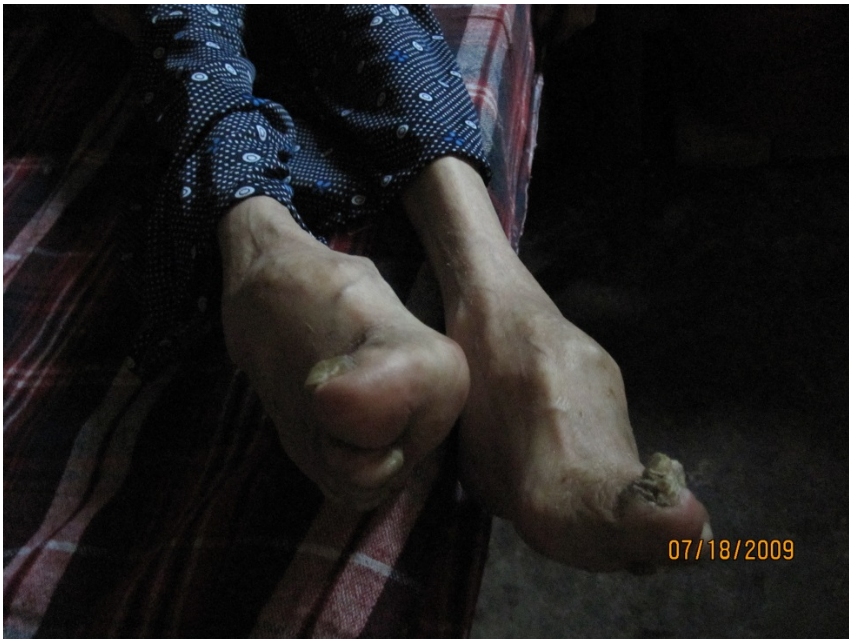

A.

Fig A. The "lotus" (*lian*) feet of an elderly Chinese woman. This 102-year-old woman (ID 2001100, born 1907, photographed in July 2009) was footbound as a child and throughout her life in the extreme "lotus" form. Her toes are still folded under the bottom of her feet and her arch is broken. This woman had to continue to bind her feet in order to be able to walk; she only loosened the bindings when she became bedridden in her 90s.

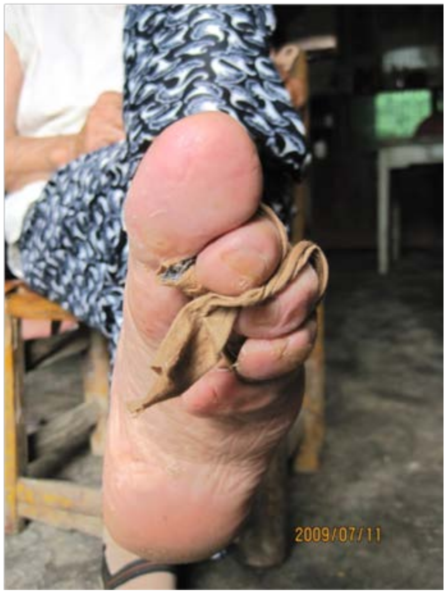

B.

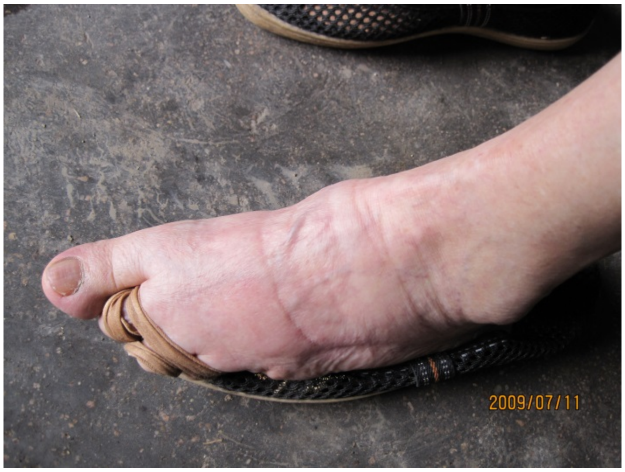

C.

Figs B and C. The “half-sloping” (*banpo*) foot of an elderly Chinese woman. This 87-year-old Chinese woman (ID 2001021, born 1922, photographed in July 2009) was footbound as a child and teenager in the less-extreme, “half-sloping” form. Her toes are still folded under the bottom of her feet, and she wraps the toes lightly for comfort (**B**, viewing the bottom of her foot). The arch of her foot was never broken (**C**, viewing her foot from above). This woman’s feet were “let out” (unbound) during the 1940s; she was still physically active at the time of the photograph.
