# Supplementary material for: Economic correlates of footbinding: Implications for the importance of Chinese daughters’ labor
Source: PLoS One. 2018 Sep 20;13(9):e0201337. doi: 10.1371/journal.pone.0201337 (PMC6147408; doi:10.1371/journal.pone.0201337)
Supplement: S1 Analysis — Tables A–B. (PDF) [file pone.0201337.s003.pdf]

S1 Analysis: Regional Results for the FB-Predictor Model (A3–A6)

Given the long-standing acceptance of distinct macroregions in China [*24*, *47*, *54*–*55*], we examined correlates of footbinding (FB) by region. We use the same variables described in the main text (for models A1 and A2) for each region, *except* that we divided hand labor into (i) spinning, (ii) weaving cloth, and (iii) other handcraft. A brief description of each region, model results, and interpretations follow, organized by region. Here, we discuss the differences between using the year-plus FB restriction versus using all ever-footbound women (Tables A and B).

Table A. Comparison of Adjusted r^2 and Significance Levels of the FB-Predictor Models, by Whether Women Who Were Footbound (fb) Less than a Year Were Included: China-wide (model A1) and Sichuan (model A2).

	**Model A1** China-wide (all 20 counties)		**Model A2** Sichuan (all 10 counties)[‡]	
	**no FB restriction** (*n* = 1759)	**FB ≥ 1 year** (*n* = 1485)	**no FB restriction** (*n* = 4758)	**FB ≥ 1 year** (*n* = 4567)
**Adjusted r^2**	0.599	0.652	0.297	0.323
**significant correlates**[†]				
county[††]	*	*	*	*
birth year	*** (-)	*** (-)	*** (-)	*** (-)
mother FB	***	***	NA	NA
mother any education	NS	*(-)	NS	NS
woman literate	NS	NS	NS	*
wealth indicator	**	***	***	***
any commercial hand labor	**	*	***	***

With no FB restriction, all ever-fb were included as fb. With the year-plus restriction (FB ≥ 1 year), only women fb for a year of age or more were included as fb. (All never-fb women were included regardless of FB restriction.)

***$p \leq 0.001$; ** $p \leq 0.01$; * $p \leq 0.05$; NS means $p > 0.05$; (-) indicates negative correlation; NA means the variable was not tested in the model.

Notes to Table A:

[†] Only significant correlates are listed in Table A. Variables tested that were not signification in models A1 and A2 include any domestic labor and agricultural field labor.

[††] When any pairwise difference between counties shows up as significant, $p < 0.05$ for “county” overall.

[‡] Both the main and regional models for Sichuan (models A2 and A6) use all available data from all 10 counties in Sichuan. In the main model (A2), spinning, weaving (cloth), and other handicraft work are combined into a single “handicraft labor” variable.

Table B. Comparison of Adjusted r^2 and Significance Levels of the FB-Predictor Models, by Whether Women Who Were Footbound (fb) Less than a Year Were Included: Regional models A3–A6.

model	**Model A3** Northern sites		**Model A4** Central sites		**Model A5** Southwest sites		**Model A6** Sichuan sites[‡]	
FB restriction?	no (*n*=777)	≥ 1 year (*n*=676)	no (*n*=932)	≥ 1 year (*n*=856)	no (*n*=353)	≥ 1 year (*n*=304)	no (*n*=4773)	≥ 1 year (*n*=4582)
**Adjusted r^2**	0.605	0.654	0.477	0.500	0.523	0.611	0.299	0.325
**significant correlates**								
county[††]	*	*	*	*	*	*	*	*
birth year	***(-)	***(-)	***(-)	***(-)	***(-)	***(-)	***(-)	***(-)
any agricult. field labor	NS	*(-)	NS	NS	NS	NS	NS	NS
mother FB[‡‡]	*	**	***	**	***	**	NA	NA
mother any education	NS	NS	NS	NS	*(-)	**(-)	NS	NS
woman literate	NS	NS	NS	NS	NS	NS	NS	*
wealth index	*	**	NS	NS	NS	NS	***	***
any commercial spinning labor	*	*	NS	NS	NS	NS	***	***
any domestic spinning labor	NS	NS	NS	NS	**	*	NS	NS
any commercial weaving labor	NS	NS	**	NS	NS	NS	NS	NS
any domestic weaving labor	NS	**	NS	**	NS	NS	NS	*
other commercial hand labor	NS	NS	NS	NS	*(-)	NS	*	*
other domestic hand labor	NS	NS	NS	NS	NS	*	NS	NS

With no FB restriction, all ever-fb were included as fb. With the year-plus restriction (FB ≥ 1 year), only women fb for a year of age or more were included as fb. (All never-fb women were included regardless of FB restriction.)

***$p \leq 0.001$; ** $p \leq 0.01$; * $p \leq 0.05$; NS means $p > 0.05$; (-) indicates negative correlation; NA means the variable was not tested in the model.

Notes to Table B:

[††] When any pairwise difference between counties shows up as significant, $p < 0.05$ for "county" overall.

[‡] Both the main and regional models for Sichuan (models A2 and A6) use all available data from all 10 counties in Sichuan. In the main model (A2), spinning, weaving (cloth), and other handicraft work are combined into a single "handicraft labor" variable. In the regional model (A6), they are three separate variables.

[‡‡] This variable did not exist in the Sichuan dataset.

Northern region

In the Northern region (Fig 4 in the main text), 10 of our 11 counties fall within one of China's winter-wheat zones, which are dry both summer and winter [*7*, *29*]. During the early 20th century, winter wheat was grown with sorghum in the low-lying and flood-prone eastern part of the region, while millet was grown in the western part where elevations were over 1000 meters. Cotton grew well in the southwestern and eastern parts of this Northern region [*7*]. County 1102 falls into the wetter Yangzi rice-wheat zone, but we classified it in our Northern region because it fell inside Skinner's North China region [*24*]. By 1936, this Northern region had more

railroads, a greater density of urban areas, and more textile mills than the other regions in our study (compare Figs 3–6), but especially in the west, most village transport was by carrying or draft animal (few roads could accommodate carts) [*7, 51*].

In model A3 to identify FB correlates in the Northern region, we found county, birth-year, wealth, whether mother was footbound (fb), and a commercial handicraft all to be significant, and knowledge of FB prohibitions *not* to have significance, all in agreement with the larger China-wide model (A1; see Tables A and B above). Domestic hand labor was predominant (almost 90 percent of women); 45 percent did commercial hand labor, and 7.8 percent reported no hand labor (Table 3). Girls performing commercial spinning were more likely to have been fb (p = 0.018), girls from wealthier natal families were more likely to have been fb (p =0.003), and girls whose mothers were fb were more likely to have been bound (p = 0.010). In addition, agricultural field labor and a domestic handicraft, which were not significant in the China-wide model (A1), were significant in the Northern region (model A3) with the year-plus restriction applied to the dataset. Girls who wove domestically before marriage were more likely to be fb (p = 0.005), and women who did crop labor in their natal households were *less* likely to be footbound than those who did no crop labor (p = 0.035). These latter two variables, however, were not significant FB correlates when the year-plus restriction was removed so that all ever-fb women were included.

It is particularly important that knowledge of prohibitions did *not* correlate to an absence of footbinding (neither ever-footbinding, p=0.296; nor footbinding for a year of age or more, p=0.260; *26*), despite the extent of anti-FB efforts in this region. 75.3 ± 3.9 percent of Northern women who married before 1950 (n=461) had heard of such prohibitions as girls (before the founding of the PRC). Many mentioned warlords Yan Xishan or Feng Yuxiang by name when

asked about prohibitions (e.g., IDs 2902088, 1901033; see also [*23*]), yet 66.7 (± 4.1) percent of Northern women married before 1950 (n=496) were ever fb and 41.9 (± 4.3) percent were fb a year or more. That is, 86.0 (± 4.4) percent of Northern ever-fb women were bound for a year or more (n=242).

It is also noteworthy that commercial spinning was still important in the Northern region with so many 1930s-era textile mills and such an expanse of railroad (Fig. 4), because it emphasizes the relative economic isolation of Northern rural villages. Girls could still make economic contributions to their families by commercial spinning [*23*, *42*, *51*, *53*]. This result makes the significance of domestic weaving, and not commercial weaving, somewhat puzzling. As mentioned in the main text, the domestic correlation could reflect erroneous reporting of production for direct exchange between households (a commercial use) as domestic use. Such underreporting applies particularly to the Northern region because we know that such misreporting occurred extensively in Anhui county 1101. However, it is also possible that, in that time and place, handspun thread was primarily being bought by those weaving for domestic use (rather than weavers intending to sell or exchange the cloth they made). Because the production of cloth required much more time spinning (using a single-spindle spinning wheel) than weaving (using a simple wooden-frame shuttle loom [*13*, *42*]), even weavers with multiple spinners in the household often needed to purchase thread, so domestic weavers may have been creating the commercial demand for homespun thread.

In any case, families benefited substantially from domestic weaving, by saving the cost of clothing and bedding [*14*]. According to Gates [*21*], missionary Adele Fielde—writing during the late 19th century about Fujian Province on China's much warmer Southeast Coast where the mean January temperature circa 1930 was 14.8^{0}C [*7*]—calculated the value of all clothing and

bedding as equivalent to the cost of all agricultural tools and resources, except the land itself. In the Northern climate, where the mean January temperature circa 1930 was 1.6^{0}C [*7*], we can expect that cost—and hence the savings from domestic weaving—would be greater.

Central region

In the Central region, 4 sites are located in the rice-tea zone, a wetter environment lying below 1000 meters [*7*, *29*]. These 4 sites, on China's central plains, enjoyed relatively easy water transport during the early 20th century, with county 2302 on the Yangzi River itself, one of China's major east-west transport lines (Fig 5). County 2001 is located in the wet Yangzi rice-wheat zone, against the low mountains separating the Middle Yangzi region from North China; it was two day's walk from the major urban center of Wuhan during the 1940s. Cotton grew at all the Central counties except county 2002, in the mountainous Sichuan rice zone, where maize (grown on slopes) was the main staple crop and dry-field rice grew on flat patches. Long-distance traders interviewed said that it took 4–5 days to cross into Sichuan to market local goods, but it was an easier journey than dropping 1500 meters in elevation to the city of Yichang. There were four major urban centers with large textile mills in the Central region, and one medium-distance trader from county 2302 reported a small mill in the city of Fuzhou, Jiangxi Province in existence by 1919. Fuzhou was a day and a half journey, using a combination of small water transport and walking, which made bringing factory-produced cloth back from Fuzhou expensive enough that it was usually reserved for wedding clothes.

In model A4 to identify FB correlates in the Central region, we found county, birth-year, and mother's FB status to be significant, and knowledge of FB prohibitions was *not* significant

(ever-fb *p*=0.869; fb-1-yr *p*=0.308; *26*), as in the Northern region (model A3) and China-wide (model A1; see Tables A and B above). Girls whose mothers were fb were more likely to be fb (p = 0.002). However, in a departure from models A1 and A3, wealth was *not* a significant correlate of FB. As in the Northern region, domestic hand labor was again predominant: 81.2 percent of women did domestic hand labor, 28 percent commercial, and 16.9 percent neither (Table 4). When the year-plus restriction was applied, Central-region domestic weavers were significantly more likely to be fb (p = 0.001). Interestingly, when all ever-fb women were included, *commercial* weaving was a significant correlate rather than domestic weaving (Table B above).

Again, it is important that knowledge of prohibitions did not correlate with an absence of FB, despite the fact that much of this region was controlled by the Nationalist government prior to Japan's 1937 invasion of China. Nationalist efforts at anti-FB prohibitions were apparently less effective than those of the Northern warlords, since only 60.3 (± 5.6) percent of Central women (married before 1950: *n*=295) reported they had heard of FB prohibitions as girls. Even in county 2302, which was close enough to a town where Nationalist troops were quartered before 1937 that people reported soldiers stealing from them, only 53.3 (± 11.3) percent of women had heard of FB prohibitions (*n*=75). Fewer women may have heard of FB prohibitions in the Central than in other regions because FB ended earlier here (Figs 7–10): only 45.9 ± 5.4 percent of Central women (married before 1950: *n*= 329) were ever-fb and only 25.7 ± 5.0 percent were fb for a year or more (*n*=292). That is, 65.8 (± 8.7) percent of Central ever-fb women were bound for a year or more.

The importance of *domestic* weaving along with the lack of importance of wealth as correlates to FB may reflect the greater commercialization of the Central region than other

regions in our sample [*7*]. It may also reflect an underreporting of weaving for direct exchange of goods as commercial (there is limited qualitative evidence suggesting that some underreporting occurred). Moreover, given mean January temperatures of 5.3^{0}C in the rice-tea zone and 3.8^{0}C in the Yangzi rice-wheat zone [*7*], domestic weaving provided real savings to household economies on the costs of clothing and bedding. However, that *commercial* weaving was significant when the year-plus FB restriction was removed suggests that the difference in the likelihood of being fb related to whether a girl was doing commercial or domestic weaving is small and there are too few data points to detect that small difference.

Southwest region

In the Southwest region (Fig 6), all counties fall within the southwest rice zone, which had elevations of 2000 meters or more and grew rice on the flat lands, especially alluvial plains and near lakes, and grew maize and opium poppy on the hillsides; cotton had to be imported [*7, 29, 41*]. The only major city in the region, Kunming, was not an industrial center prior to World War II (which began in 1937 in East Asia).

In model A5 to identify FB correlates in the Southwest region, as in the China-wide and other regional models (A1, A3, A4), we found county, birth-year, and whether mother was fb to be significant, and knowledge of FB prohibitions *not* significant (ever-fb p=0.398; fb1yr p=0.311). Mother's education was significant, as in the China-wide model (A1), though it was not in other regional models (A3, A4). Mothers with at least some education were less likely to have fb daughters (p = 0.004). As in the Central region (model A4), wealth was *not* a significant correlate of FB. In the Southwest region, *domestic* handicraft production was again significant:

both domestic spinning and our domestic “other” category, which includes all handicrafts other than spinning and weaving cloth. In the Southwest, domestic hand labor still predominated (83.6 percent of women), but commercial hand labor was much more prevalent here than in any other region (Sichuan included): 64.1 percent of women reported doing commercial hand labor (and only 9.8 percent did no hand labor; Table 5).

It is important that knowledge of prohibitions again did not correlate with absence of FB. Nationalist prohibitions of FB were better known here than in the Central region: 70.7 (± 7.3) percent of Southwest women (married before 1950, *n*=150) reported hearing of FB prohibitions as girls, and many women referred to household inspections and fines if FB was discovered. Nevertheless, FB lingered long here: 79.4 (± 6.4) percent of Southwest women (married before 1950; *n*=155) were ever-fb and 50.0 (± 9.0) percent were fb for more than a year. That is, 68.6 (± 9.8) percent of SW ever-fb women were bound for a year or more.

It is unclear why it is (again) *domestic* handicraft production that is significant, unless it reflects underreporting of direct exchange as commercial. Moreover, with the year-plus restriction, domestic spinning was a positive correlate of FB (p = 0.018), as was our “other” domestic handicrafts category (p = 0.041). Without that restriction (i.e., with all ever-fb women included), other *commercial* handicrafts were a *negative* correlate of FB (p = 0.035): girls doing commercial handicrafts other than spinning and weaving cloth were *less* likely to be fb than girls not doing other commercial handicrafts. Domestic spinning continued to be a positive correlate (p = 0.008).

Sichuan region

The 10 Sichuan counties all fall within the Sichuan rice zone, a basin close to 1000 meters elevation nestled in mountains that begin the approach to the Himalayas [*7, 29*]. The Sichuan counties span the range of accessibility and commercialization during the early 20th century, from Sichuan county MS (Mingshan), which was mountainous and remote, to counties BX (Ba Xian) and LQ (Longchuanyi Qu), which were rural hinterlands of the river-port city of Chongqing and the provincial capital, Chengdu, respectively (Fig 3). Although somewhat drier than the southwest rice zone, the Sichuan rice zone also grew rice, maize, and opium poppy and had to import cotton [*7*]. Prior to WWII, neither Chongqing nor Chengdu were major industrial centers.

Treating Sichuan as a region in which to identify correlates of FB (model A6)—by which we mean that hand labor was broken down into spinning, weaving cloth, and other handcrafts—conformed to the China-wide model (A1) and the main Sichuan model (A2, where different types of handicrafts were not distinguished). We found county, birth-year, wealth, and commercial handicraft production to be significant regardless of whether the year-plus restriction was included or not, and an educational measure to be significant when the year-plus FB restriction was included. (As in the Sichuan main model [A2], this regional Sichuan model [A6] does not consider whether a woman’s mother was fb or whether she had heard of FB prohibitions as a girl as correlates, since these data were not collected in Sichuan.) Interestingly, in Sichuan, commercial hand labor predominated (43.6 percent of women) over domestic hand labor (32.7 percent), but as many as 37 percent of women reported no hand labor at all (Table 6). Two kinds of commercial handicraft production were significant (regardless of whether the year-plus restriction was included): commercial spinning ($p < 0.001$) and “other” commercial handicraft

labor (*p* = 0.022). Additionally, as in the Northern and Central regions (models A3 and A4), domestic weaving was significant (p = 0.033) when using the year-plus FB restriction, but not significant when the restriction was not used (Table B above).

As in the main Sichuan model (A2), a woman's literacy level was significant when using the year-plus FB restriction: illiterate girls were more likely to have been bound than literate ones (*p* = 0.024). (There was no one in the half-literate category in the sample of the Sichuan data that met all our restrictions and had information for each of the examined variables.) Among the regional models, only the Southwest (model A5) and Sichuan (model A6) had a significant educational correlate (and only in the Southwest was it significant regardless of whether the year-plus FB restriction was included; Table B above).

The importance of commercial spinning and other commercial handicraft in early 20th-century Sichuan, prior to industrialization and for a sample of women born 1887–1930, suggests that the relationship between FB and commercial handicraft production may extend further back into China's late imperial period. This regional finding further supports the result of our generational model (B1) that women's mothers, born 1882–1931, were more likely to be spinners if they were fb. The significance of domestic weaving, and not commercial weaving, in the Sichuan regional model (A6) may again reflect underreporting of cloth exchange as commercial. In any case, as in the Northern and Central regions, domestic weaving must have provided important savings on the costs of clothing and bedding in Sichuan, where the mean January temperature circa 1930 was 7.0^{0}C [*7*].
