# Supplementary material for: Economic correlates of footbinding: Implications for the importance of Chinese daughters’ labor
Source: PLoS One. 2018 Sep 20;13(9):e0201337. doi: 10.1371/journal.pone.0201337 (PMC6147408; doi:10.1371/journal.pone.0201337)
Supplement: S2 Analysis — Tables A–B. (PDF) [file pone.0201337.s004.pdf]

S2 Analysis: County Results for the Daily-Labor Model (C2–C5)

To explore correlates of the daily quantity of cotton thread produced at the level of individual counties, we examined 4 counties where 25 or more women (who married before 1950) reported the amount of cotton they spun per day. Note that the numbers drop below 25 when other data restrictions were imposed. We ran models C2–C5 as described in the main text, except that we removed “county” as a variable. Significant correlates of quantity produced, adjusted r^2 values, and predicted values of daily amount of cotton spun are reported in terms of county, data restrictions imposed, and number of data points (Tables A and B below). Throughout this analysis, when we refer to the “predicted” or “fitted daily amount spun,” we refer to the fitted value at 12 *sui* (about 11 years old).

Table A. Comparison of the Daily-Labor Model Results China-wide (model C1) and in Four Counties (models C2–C5), by Whether Women Who Were Footbound (fb) Less than a Year Were Included: Adjusted r^2 and significance levels.

model	**C1. China-wide** (14 counties)		**C2. site 2001** (in Central)		**C3. site 2102** (in Central)‡		**C4. site 2302** (in Central)		**C5. site 2902** (in North)‡	
FB restriction?	no n=153	≥ 1 yr n=137	no n=16	≥ 1 yr n=15	no n=26	≥ 1 yr n=19	no n=14	≥ 1 yr n=13	no n=27	≥ 1 yr
**Adjusted r^2**	0.122	0.142	0.123	0.324	0.249	0.181	0.412	0.425	0.442	NA§
**significant correlates†**										
county††	*	*	NA	NA	NA	NA	NA	NA	NA	NA
commercial spinning	***	*	NS	NS	NS	NS	NS	NS	NS	NA

domestic weaving or other hand labor	NS	NS	NS	NS	NS	NS	NS	NS	*(-)	NA
woman ever fb	NS	NS	NS	NS	*	NS	NS	NS	NS	NA
woman any education	NS	NS	NS	NS	**(-)	NS	NS	NS	**	NA
age learned to spin‡	NS	NS	NS	NS	NS	NS	*	*	***(-)	NA
family loom	NS	NS	NS	NS	NS	NS	NS	NS	***(-)	NA
**households growing cotton**	36.4%		36.4%		44.8%		40.1%		61.2%	

With no FB restriction, all ever-fb were included . With the year-plus restriction (FB ≥ 1 year), only women fb for a year or more were included . (All never-fb women were included in all models.)

*** ≤ 0.001; ** ≤ 0.01; * ≤ 0.05; NS means p > 0.05; (-) indicates negative correlation, for binary variables yes=1, no=0; NA means the variable was not tested in the model. Unless otherwise stated, the predicted values use the median age of learning to spin: 12 *sui* or approximately 11 years old.

† Only variables that had significance in one or more models are reported.

†† When any individual county shows up as significant, $p < 0.05$ for "county" overall.

‡For model C3, the correlate is positive and the transforming exponent in the model is negative. For model C5, the correlate is negative and the transforming exponent in the model is positive. Thus, for both models, the younger the age at which a woman learned to spin, the more cotton that she spun per day just before she left her natal household for marriage.

§ The year-plus restriction was not run at site 2902 because these 27 women did not provide information allowing calculation of footbinding duration.

Table B. Comparison of the Daily-Labor Model Results China-wide (model C1) and in Four Counties (models C2–C5), by Whether Women Who Were Footbound (fb) Less than a Year Were Included: Predicted values (in grams) of the daily amount of cotton spun by an average girl.

model	**C1. Chinawide** (14 counties)		**C2. site 2001** (in Central)		**C3. site 2102** (in Central)[‡]		**C4. site 2302** (in Central)		**C5. site 2902** (in North)[‡]
FB restriction?	no *n*=153	≥ 1 yr *n*=137	no *n*=16	≥ 1 yr *n*=15	no *n*=26	≥ 1 yr *n*=19	no *n*=14	≥ 1 yr *n*=13	no[‡‡] *n*=27
**Adjusted r^2**	0.122	0.142	0.123	0.324	0.249	0.181	0.412	0.425	0.442
**in the presence of significant correlates**									
no significant correlates			207.3	207.2		175.7			
commercial spinning = no	97.0	96.2							
commercial spinning = yes	148.9	138.6							
FB=no, educ=none					259.8				
FB=no, educ=some					202.6				
FB=yes,educ=none					319.5				
FB=yes, & education=some					251.5				
age at learning to spin = 8 *sui*							171.7	177.3	247.8
age at learning to spin = 12 *sui*							119.3	119.3	160.1
age at learning to spin = 18 *sui*							61.9	68.2	70.6
family loom = yes									84.8
woman's education=some[§]									249.7

weaving or other hand labor = yes									122.2
**households growing cotton**	36.4%		36.4%		44.8%		40.1%		61.2%

With no FB restriction, all ever-fb were included. With the year-plus restriction (FB ≥ 1 year), only women fb for a year or more were included. (All never-fb women were included in all models.) Unless otherwise stated, the predicted values use the median age of learning to spin: 12 *sui* or approximately 11 years old.

Notes to Table S2:

† Only variables that had significance in one or more models are reported.

†† When any individual county shows up as significant, $p < 0.05$ for "county" overall.

‡For model C3, the correlate is positive and the transforming exponent in the model is negative. For model C5, the correlate is negative and the transforming exponent in the model is positive. Thus, for both models, the younger the age at which a woman learned to spin, the more cotton that she spun per day just before she left her natal household for marriage.

‡‡ For site 2902, with no FB restrictions, there were four significant correlates (age at learning to spin, family loom, woman's education, and weaving or other hand labor). Quantities in this column show all correlates as "no" except for the single correlate in that row. For example, the correlates in the row "age at learning to spin = 12 *sui*" are: age at learning to spin = 12 *sui*, family loom = no, woman's education = none, weaving or other hand labor = no. The year-plus restriction was not run at site 2902 because these 27 women did not provide information allowing calculation of footbinding duration.

§ In C3, both education and the education:FB interaction were significant; in C5 only education was significant, hence the appearance of education as a separate variable here.

Hubei County 2001 (model C2)

In county 2001 (model C2), in our Central region, with only 15–16 women who provided data on all the necessary variables, we found no significant correlates of daily production quantity

(regardless of whether the year-plus FB restriction was applied). The predicted value of cotton spun daily was 207 grams.

Hunan County 2102 (model C3)

In county 2102 (model C3), in our Central region, finding any of the correlates significant varied with the FB restriction. Note that using the year-plus FB restriction reduced the number of women with data for all the variables. When all ever-fb women were included (n=25) (i.e., there was no restriction), both FB and educational status were significant: ever-fb girls spun more cotton per day than never-fb girls (p=0.036), and girls with no education spun more than girls with some education (p=0.006). Moreover, predicted values of daily cotton spun by girls with one or both of these significant variables were *much* higher than the 217 grams spun daily by enslaved African American girls at Thomas Jefferson's plantations: 252 grams for girls who were footbound and had some education, 260 grams for never footbound and no education, and 320 grams for footbound with no education. With the year-plus FB restriction imposed (n=19), there were no significant variables, and the predicted value of cotton spun by the average girl dropped to 176 grams. This is the only occasion where we found the year-plus FB restriction to yield a *poorer* goodness of fit (adj. r^2=0.181) than using all ever-fb women without restriction (adj. r^2=0.249). These differences are almost certainly due to small sample size.

Jiangxi County 2302 (model C4)

At county 2302 (model C4), in our Central region, with only 13–14 women providing data, there was a single significant correlate of daily production: the age at which a girl learned to spin (p =

0.011). This result obtained regardless of whether imposing the year-plus FB restriction. The younger a girl's age when she learned to spin, the more she spun per day (at the end of her time in her premarital household). Predicted values of daily quantity (at age=12 *sui*) range from 177 grams, for someone who learned to spin at 8 *sui*, to 68 grams, for someone who learned at 18 *sui*. Girls who learned at the median age of 12 *sui* (about 11 years old) have a predicted daily value of 119 grams.

Shanxi County 2902 (model C5)

At county 2902 (model C5), in our Northern region, it was not possible to apply the year-plus restriction because so few women reported the age at which they were fb that, once other restrictions on the data were included (e.g., married before 1950), there were not sufficient data available on how long women's feet were bound. Using all ever-fb women, we found 4 significant correlates: the age at which a girl learned to spin, whether a woman's premarital family had a loom, whether the woman had some education prior to 1950, and whether a girl did any domestic handicraft other than spinning (Table B above). Here, as in county 2302 (model C4), the younger a girl's age when she learned to spin, the more she spun per day. Unexpectedly, girls with some education spun *more* than girls with no education ($p = 0.008$). The remaining variables were both negative correlates: girls spun *less* if their families had a loom ($p < 0.001$) and if they were doing a domestic handicraft other than spinning (weaving cloth, or any other handicraft; $p = 0.045$). These results are suggestive that at this site, in families with looms, girls specialized in weaving, presumably acquiring cotton by direct exchange or purchase. Predicted values of daily quantity vary by whether girls had any or more than one of these significant variables. A girl's education and whether her family had a loom were comparable, as correlates,

to the age at which a girl learned to spin and were more important than whether a girl did any domestic handicraft other than spinning. Using the median age at learning to spin of 12 *sui* (about 11 years old), a girl with none of the other significant variables (no education, no family loom, and no other domestic handicrafts) had a predicted value of 160 grams per day. A comparable girl (no education, no family loom, and no other domestic handicrafts) who learned to spin at 8 *sui* (about 7 years old) had a predicted value of 248 grams per day, while a comparable girl who learned to spin at 18 *sui* (about 17 years) had a predicted value of 71 grams. A girl with some education but none of the other significant variables (no family loom, no other domestic handicrafts, and who learned to spin at the median age of 12 *sui*), had a predicted value of 250 grams per day, while a girl with a family loom (but no education, no other domestic handicrafts, and who learned to spin at the median age) had a predicted value of 85 grams per day. (See Appendix C for a discussion of the implications of these county-level results.)
